# Neuroprotective effect of a medium-chain triglyceride ketogenic diet on MPTP-induced Parkinson’s disease mice: a combination of transcriptomics and metabolomics in the substantia nigra and fecal microbiome

**DOI:** 10.1038/s41420-023-01549-0

**Published:** 2023-07-17

**Authors:** Wenlong Zhang, Shiyu Chen, Xingting Huang, Huichun Tong, Hongxin Niu, Lingli Lu

**Affiliations:** 1 Department of Neurology, The First Affiliated Hospital of Guangzhou Medical University, Guangzhou, Guangdong Province 510120 China; 2 Department of General practice, Zhujiang Hospital, Southern Medical University, Guangzhou, Guangdong Province 510282 China; 3 Guangdong Key Laboratory of Non-human Primate Research, Guangdong-Hongkong-Macau Institute of CNS Regeneration, Jinan University, Guangzhou, Guangdong Province 510632 China; 4 General practice and Special medical service center, Zhujiang Hospital, Southern Medical University, Guangzhou, Guangdong Province 510282 China

**Keywords:** Parkinson's disease, Neural ageing

## Abstract

The ketogenic diet (KD) is a low carbohydrate and high-fat protein diet. It plays a protective role in neurodegenerative diseases by elevating the levels of ketone bodies in blood, regulating central and peripheral metabolism and mitochondrial functions, inhibiting neuroinflammation and oxidative stress, and altering the gut microbiota. However, studies on ketogenic therapy for Parkinson’s disease (PD) are still in their infancy. Therefore, we examined the possible protective effect of KD in a 1-methyl-4-phenyl-1,2,3,6-tetrahydropyridine (MPTP)-induced PD mouse model, examined the mouse gut microbiota and its metabolites, and performed transcriptomics and metabolomics on the substantia nigra of mice. Our results showed that a long-term medium-chain triglyceride KD (MCT-KD) significantly reduced MPTP-induced damage to dopaminergic (DA) neurons, exerted antioxidant stress through the PI3K/Akt/Nrf2 pathway, and reversed oxidative stress in DA neurons. The MCT-KD also reduced mitochondrial loss, promoted ATP production, and inhibited the activation of microglia to protect DA neurons in MPTP-induced PD mice. MCT-KD altered the gut microbiota and consequently changed the metabolism of substantia nigra neurons through gut microbiota metabolites. Compared to the MPTP group, MCT-KD increased the abundance of gut microbiota, including Blautia and Romboutsia. MCT-KD also affects purine metabolism in the substantia nigra pars compacta (SNpc) by altering fecal metabolites. This study shows that MCT-KD has multiple protective effects against PD.

## Introduction

Parkinson’s disease (PD) is the second most common movement neurodegenerative disease of the central nervous system, affecting 3.9‰ and 1–2‰ of individuals in China and worldwide, respectively [1]. The cardinal motor symptoms of PD result from the loss of dopaminergic (DA) neurons in the nigrostriatal system [2]. The pathogenesis of PD is multifactorial, including mitochondrial dysfunction, the misfolding and aggregation of proteins, endoplasmic reticulum stress, dysfunction of the autophagy-lysosome system, and neuroinflammation [3]. Additionally, there is some evidence that the energy metabolism of DA neurons is impaired [4, 5]. Due to the long and highly branched axon and multitudinous synaptic sites, DA neurons have a high energy demand [6–8] and autonomous pacemaking activity, highlighting their vulnerability to energy deprivation [6, 9]. ^31^P-MRS and ^18^FDG-PET imaging has shown that both glycolytic and oxidative pathways were impaired in non-demented patients with PD [10]. Therefore, using a more energy-efficient and stable fuel may improve PD symptoms.

A high-fat, low-carbohydrate, and moderate protein diet, known as a ketogenic diet (KD), has been used as a non-pharmacological treatment for refractory epilepsy [11]. Increasing numbers of studies have shown that the KD has protective effects on neurodegenerative changes such as Alzheimer’s disease, Parkinson’s disease, epilepsy, and stroke [12]. However, few in vivo experimental studies related to PD have been published [13–21]. The specific mechanism by which the KD protects DA neurons is unclear. Ketone bodies are the natural alternative substrates to glucose for cerebral energy metabolism [22]. When the glucose supply is insufficient, ketones can directly enter the TCA cycle to bypass the glycolysis pathway, thus providing a more efficient energy source [23–25]. Ketone bodies not only improve mitochondrial metabolism but also inhibit ROS/superoxide production to reduce oxidative stress [26], which is coupled with anti-inflammatory effects [27]. Compared to the conventional KD, KD dominated by medium-chain triglyceride KD (MCT-KD) can produce more ketones and reduce the adverse effects of ketone induction [28, 29]. The MCT-KD can cross the blood-brain barrier and act as an alternative energy source for neurons and astrocytes [9]. However, current research on the use of a KD for treating PD mainly involves long-chain fatty acids, while research on the use of MCT-KD is lacking [16–19]. Therefore, the function of the MCT-KD in protecting DA neurons remains to be confirmed.

The gut microbiota are the key mediators of the physiological connection between the host and diet, and the species composition and function of gut microbiota are affected by diet [30, 31]. KD also alters gut microbiota [30]. Diet-induced changes in the gut microorganisms are replicable and persistent, and as such, have lasting impacts on the host [32]. The influence of the gut microbiota is profound, affecting nerve development, behavior, neurotransmitter production, and inflammation [33]. The gut microbiota may also affect the nervous system through the release of metabolites [34]. Recently, several studies have demonstrated that patients with PD have gut dysbacteriosis and emphasize the effect of the gut microbiota on the brain–gut axis [35, 36]. Although emerging evidence links dysbacteriosis to the pathogenesis of PD, there is little research on improving symptoms by changing gut microbiota through the diet. The effects of gut microbiota changes caused by a long-term MCT-KD on PD require further research.

In this study, MCT-KD was used as a strategy for PD therapy in a MPTP-induced PD mouse model. In vivo and in vitro studies have shown that MCT-KD directly enhanced the antioxidant stress capacity of DA neurons by increasing ketone body levels in mice. MCT-KD also reduced the activation of proinflammatory microglia. In addition, 16 S rRNA sequencing and metabolomics analysis confirmed that MCT-KD altered the gut microbiota and its metabolites and influenced the purine metabolism of cells in the substantia nigra, indicating that MCT-KD has great potential for neuroprotection against PD.

## Results

### MCT-KD improved motor deficits and protected DA neurons in PD mice

**Fig. 1:**
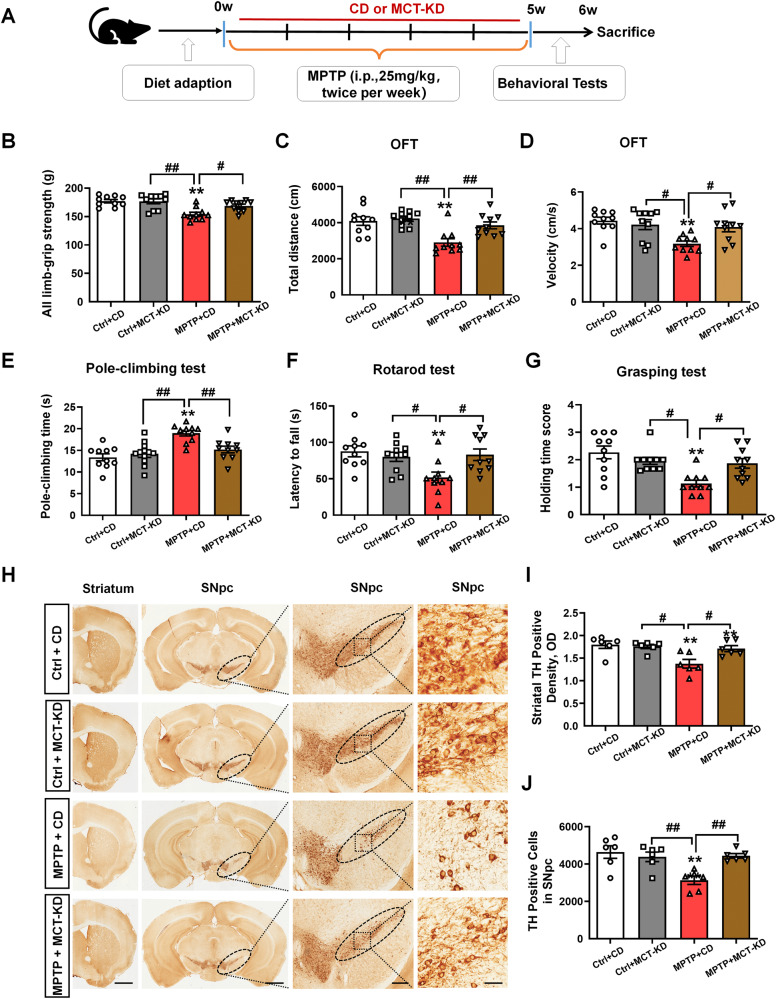
MCT-KD improved motor deficits and protected DA neuron loss in PD mice. **A** Experimental design for medium-chain triglyceride KD (MCT-KD) treatment in MPTP-induced PD mice. **B** The limb-grip strength test was used to measure the muscle strength of the forelimbs. **C** Total travel distance of the mice in the open field experiment. **D** Mean velocity of the mice in the open field experiment. **E** The pole-climbing test was used to assess the motor coordination of mice. **F** The rotarod test was used to assess the motor coordination of mice. **G** The grasping test was used to assess the grip strength of mice; *n* = 10 for each of the Ctrl + CD group, Ctrl + MCT-KD group, MPTP + CD group, and MPTP + MCT-KD group. **H** Immunohistochemical staining of TH-positive neurons in the striatum and pars compacta of substantia nigra (SNpc) in the Ctrl + CD group, Ctrl + MCT-KD group, MPTP + CD group, and MPTP + MCT-KD group. The ellipse represents the boundary of the SNpc, and the middle box represents the area expanded in the right column. **I** Quantification of the TH-positive density in the striatum. **J** Quantification of TH-positive neurons in the SN; *n* = 6. Data are presented as the mean ± SEM. **p < 0.01 vs. the Ctrl + CD group. ^##^*p* < 0.01, ^#^*p* < 0.05 vs. the MPTP + CD group. One-way ANOVA with Tukey’s post hoc analysis was used for comparison among multiple groups.

We employed an MPTP-induced chronic PD mouse model to evaluate whether the MCT-KD confers neuroprotection in DA neurons [37]. MPTP was administrated intraperitoneally continuously twice a week over a 5 weeks period to induce the chronic PD model (Fig. 1A). Compared with the control (Ctrl) + control diet (CD) group, the MPTP + CD group suffered severe motor ability damage (Fig. 1B–G). We found that the MCT-KD reversed the MPTP-induced decrease in limb-grip strength; the total distance traveled and mean velocity using the open field test; the decrease in latency to fall using the rotarod test; and the decrease in holding time score using the grasping test, as well as the MPTP-induced increase in pole-climbing time using the pole-climbing test. There was no difference between the CD and MCT-KD groups in non-MPTP mice (Fig. 1B–G). These findings indicated that MCT-KD did not alter the behavior of non-MPTP mice. To confirm the protective effect of the MCT-KD on DA neurons, we measured the tyrosine hydroxylase (TH) levels in the striatum and substantia nigra. The MCT-KD reversed the MPTP-induced decrease in striatal TH density and TH-positive cells of SNpc (Fig. 1H–J). We also found that the MCT-KD did not influence TH density in SNpc in non-MPTP mice. No significant pathological changes were found in their heart, kidneys, lungs, liver, spleen, and colon between the Ctrl + CD group and Ctrl + MCT-KD group (sFig. 1). These results indicated that a long-term MCT-KD could protect DA neurons without affecting other organs. Combined with the above results, it can be concluded that the administration of the MCT-KD alone did not affect the behavior, DA neurons, and non-targeted organs of non-MPTP mice.

**Fig. 2:**
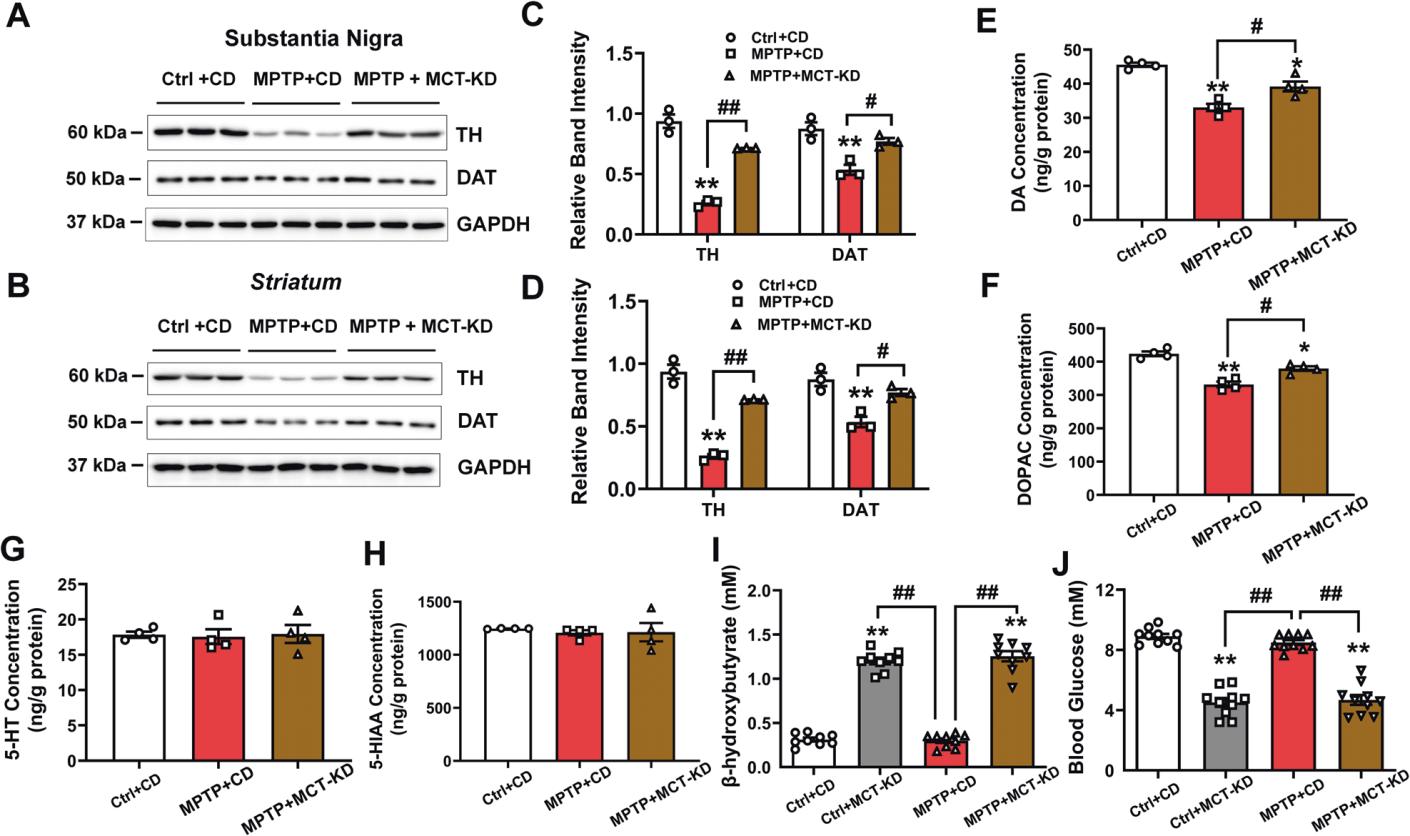
MCT-KD increased TH expression and DA synthesis in PD mice. **A**–**D** Western blots and quantitative analysis showed the expression levels of TH and DAT in the SN and striatum in the Ctrl + CD group, MPTP + CD group, and MPTP + MCT-KD group; *n* = 3 per group. **E**–**H** Expression levels of dopamine, DOPAC, 5-HT, and 5-HIAA were assessed by HPLC‐MS/MS analysis; *n* = 4 per group. **I**, **J** β-hydroxybutyrate and glucose in plasma were measured; *n* = 10. Data are presented as the mean ± SEM. **p < 0.01, *p < 0.05 vs. the Ctrl + CD group. ^##^*p* < 0.01, ^#^*p* < 0.05 vs. the MPTP + CD group^.^ Statistical significance was determined by one-way ANOVA and Tukey’s tests for post hoc comparisons.

Western blotting showed that MCT-KD increased the expression of TH and dopamine transporter (DAT) in the substantia nigra and striatum of PD model mice (Fig. 2A–D). Furthermore, MPTP decreased the concentration of DA and its metabolite [3,4-dihydroxyphenylacetic acid (DOPAC)], which was reversed by MCT-KD (Fig. 2E, F). Interestingly, there was no MPTP-induced decrease in 5‐hydroxytryptamine (5-HT, serotonin) or 5‐hydroxyindoleacetic acid (5‐HIAA, the major end‐product of serotonin metabolism) (Fig. 2G, H).

Because the MCT-KD is based on changing the energy supply of mice, we examined the level of plasma β-hydroxybutyrate and blood glucose in mice. Compared to mice fed the CD, mice fed the MCT-KD had a higher level of plasma β-hydroxybutyrate (Fig. 2I). Due to the low carbohydrate content of the MCT-KD, the blood glucose level of mice decreased significantly, which is consistent with previous studies on KDs [38, 39]. As expected, mice that were administered a long-term MCT-KD had significantly lower blood glucose than the control mice (Fig. 2J).

### MCT-KD reduced oxidative stress by upregulating the PI3k-Akt pathway

**Fig. 3:**
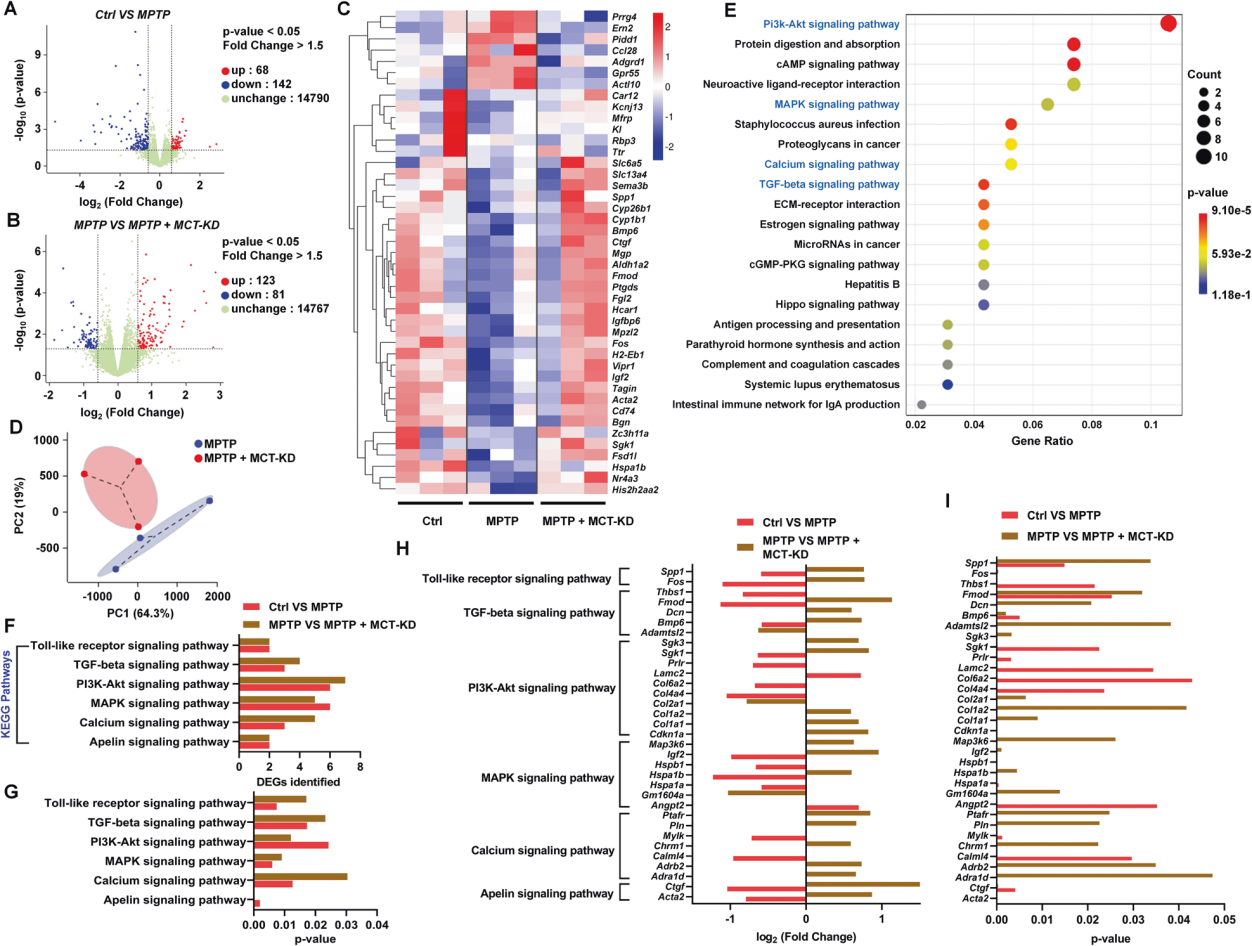
RNA-sequencing analysis of the ketogenic diet in MPTP-induced PD mouse model. **A**, **B** Volcano plots showing the differentially expressed genes (DEGs) between the Ctrl + CD group and MPTP + CD group, as well as between the MPTP + CD group and MPTP + MCT-KD group. **C** Hierarchical clustering of 43 representative MPTP + MCT-KD group reversed DEGs that were decreased in the MPTP + CD group compared to the Ctrl + CD group. **D** PCoA diagram based on the OTU matrix of mouse DEG in the MPTP + CD group and MPTP + MCT-KD group. The ellipse represents the standard deviation of the samples. **E** Kyoto Encyclopedia of Genes and Genomes (KEGG) pathways enriched by DEGs among the Ctrl + CD group, MPTP + CD group, and MPTP + MCT-KD group. Blue tones show the oxidative-stress relative pathways. **F**, **G** Number and *p*-values of identified DEGs in oxidative-stress related KEGG pathways. **H**, **I** Log2(fold change) and *p*-values of 43 individual DEGs enriched in KEGG pathways.

We next performed RNA‐sequencing analysis to identify the potential mechanisms that MCT-KD uses to protect DA neurons. Through volcano plots (Fig. 3A, B) and hierarchical clustering analysis (Fig. 3C), we found 43 representative differentially expressed genes (DEGs). Due to the interference of MPTP, the expression of these genes increased or decreased but was reversed by MCT-KD. To confirm the reliability of the analysis, we conducted principal component analysis and repeated correlation evaluation, which showed significant differences in composition between the MPTP + CD group and MPTP + MCT-KD group (Fig. 3D). We next performed KEGG analysis of these genes to further illustrate their pathway and function (Fig. 3E). The top three DEG-rich pathways were “PI3K Akt-signaling pathway,” “protein digestion and absorption,” and “cAMP-signaling pathway,” while “MAPK,” “calcium signaling pathway,” and “TGF beta signaling pathway” were also highly enriched (see blue font in Fig. 3E). For further verification, we checked the number and *p*-value of the identified DEGs of the PD-related KEGG pathway, including the “toll-like receiver signaling pathway,” “TGF beta signaling pathway,” “PI3K Akt-signaling pathway,” “MAPK signaling pathway,” “calcium signaling pathway,” and “Apelin-signaling pathway” (Fig. 3F, G). The log 2-fold changes and *p-*values of 33 individual DEGs enriched in these pathways were also measured (Fig. 3H, I).

**Fig. 4:**
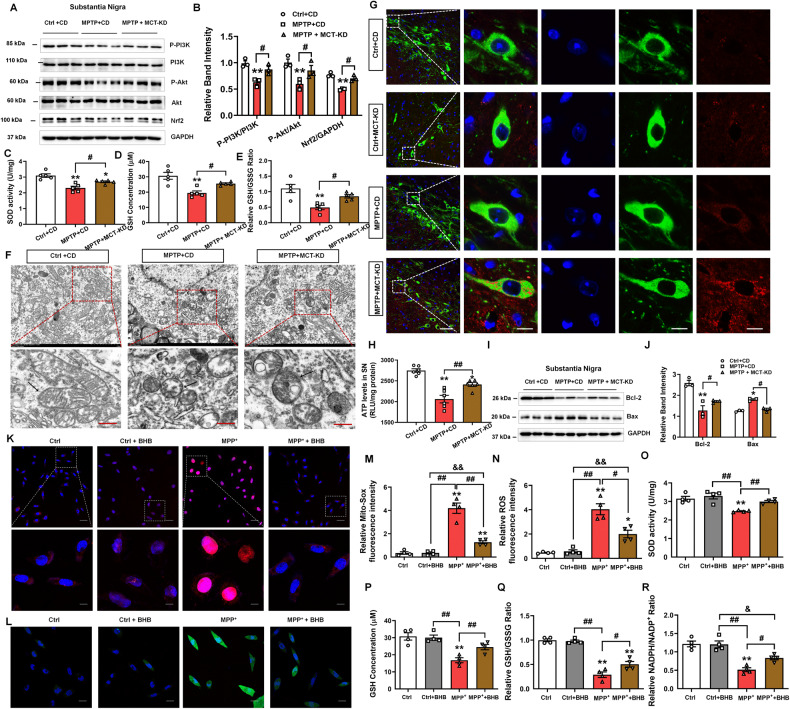
Ketogenic diet targets the PI3K-Akt pathway in the MPTP-induced PD mouse model. **A**, **B** Western blots and quantitative analysis showing the expression levels of P-PI3K, PI3K, P-Akt, Akt, and Nrf2 in the SN in the Ctrl + CD group, MPTP + CD group, and MPTP + MCT-KD group; *n* = 3 per group. **C**, **D** Contents of SOD and GSH in the three groups. **E** Relative GSH/GSSH ratio in the three groups; *n* = 5 per group. Data are presented as the mean ± SEM. **p < 0.01, *p < 0.05 vs. the Ctrl group. ^##^*p* < 0.01, ^#^*p* < 0.05 vs. the MPTP + MCT-KD group. **F** Ultrastructural analysis of mitochondria for the original and magnified images in the three groups. **G** Immunofluorescent studies of TH (green) and Bcl-2 (red) in the SN. **H** Content of ATP in SN; *n* = 6 per group. Data are presented as the mean ± SEM. **p < 0.0. vs. the Ctrl + CD group, ^##^*p* < 0.01 vs. the MPTP + CD group. **I**, **J** Western blots and quantitative analysis showed the expression levels of Bcl-2 and Bax; *n* = 3 per group. Data are presented as the mean ± SEM. **p < 0.01, *p < 0.05 vs. the Ctrl + CD group. ^##^*p* < 0.01, ^#^*p* < 0^.^05 vs. the MPTP + CD grou*p*. **K** Representative graphs of Mito-Sox (red) and Hoechst (blue) u*p*on β-hydroxybutyrate (BHB) treatment in the control or MPP^+^-treated cells. **L** Representative graphs of ROS (green) and Hoechst (blue) upon BHB treatment in the control or MPP^+^-treated cells. *n* = 4 per group. **M**, **N** Quantification of the relative Mito-Sox and ROS fluorescence intensity in the four groups; *n* = 4 per group. **O**, **P** Contents of SOD and GSH upon BHB treatment in the control or MPP + -treated cells. *n* = 4 per group. **Q**, **R** Relative GSH/GSSH and NADPH/NADP+ ratio in the control or MPP + -treated cells. *n* = 4 per group. Data are presented as the mean ± SEM. **p < 0.01, *p < 0.05 vs. the Control group. ^##^*p* < 0.01, ^#^*p* < 0.05 vs. the MPP^+^ group. ^&&^*p* < 0.01, ^&^*p* < 0.05 vs^.^ the MPP^+^ + BHB group^.^ Statistical significance was determined by one-way ANOVA and Tukey’s tests for post hoc comparisons.

To further verify the protective role of MCT-KD in PD, we used western blotting to analyze the related protein expression (Fig. 4A, B). The results showed that MPTP treatment decreased the phosphorylation of PI3K and Akt and reduced the expression of Nrf2, both of which were reversed by MCT-KD. Nrf2 is one of the downstream products of the PI3K-Akt pathway, and this product is related to antioxidant stress in neurodegenerative diseases [40]. Previous studies have indicated that a KD can prevent oxidative stress by improving mitochondrial energy metabolism [41–43]. As further validation, we evaluated the levels of oxidative stress by measuring the superoxide dismutase (SOD) activity and L-glutathione (GSH) concentration (Fig. 4C–E). Compared to the MPTP + CD group, MCT-KD increased the SOD activity, GSH concentration, and GSH/glutathione disulfide (GSSG) ratio in MPTP-treated mice. Furthermore, the TEM results indicated that MPTP led to swelling of mitochondria and the disappearance of the mitochondrial cristae, which was reversed by MCT-KD (Fig. 4F). Compared to the MPTP + CD group, the MCT-KD increased ATP levels in SN (Fig. 4H).

As oxidative stress can exacerbate neuronal apoptosis, we next detected the expression of BCL2 in SNpc. As previous results have revealed that MPTP causes mitochondrial damage, we evaluated the apoptosis signals associated with mitochondrial oxidative stress. Compared to the MPTP + CD group, the expression of anti-apoptotic protein Bcl-2 was increased in DA neurons in the MPTP + MCT-KD group (Fig. 4G). Western blotting also showed that the decrease in Bcl-2 expression caused by MPTP was reversed by MCT-KD. Compared to the MPTP + CD group, MCT-KD decreased the expression of the pro-apoptotic protein Bax (Fig. 4I, J). These results suggest that MCT-KD can prevent DA loss by regulating Bcl-2 and downregulating Bax. As further validation, we evaluated the antioxidant effects of β-hydroxybutyrate (BHB) in MPP^+^-treated DA-derived MN9D cells. Compared with the Ctrl group, Mito-Sox appeared oxidized in the MPP^+^ group. BHB treatment significantly decreased oxidized Mito-Sox induced by MPP^+^ (Fig. 4K, M). BHB also reversed the MPP+-induced ROS generation (Fig. 4L, N). Notably, BHB increased the SOD activity, GSH concentration, and GSH/GSSG ratio (Fig. 4O–Q). Additionally, BHB restored the reduced state of nicotinamide adenine dinucleotide phosphate (NADPH) and its oxidized state (NADP^+^) ratio in MPP^+^ treatment (Fig. 4R). These results suggest that BHB may reverse oxidative stress in DA neurons.

### MCT-KD alleviates gut microbiota dysbiosis in MPTP-induced PD mice

**Fig. 5:**
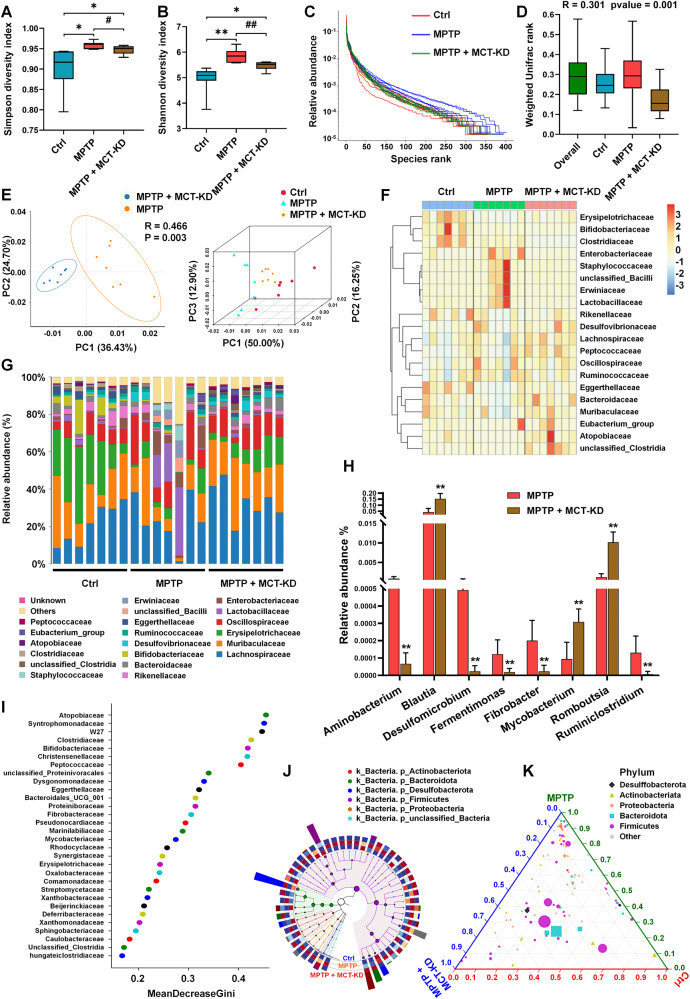
Ketogenic diet alleviates fecal microbiota dysbiosis in the MPTP-induced PD mouse model. **A**, **B** Boxplot of the Simpson diversity index (**A**) and Shannon diversity index (**B**) in the Ctrl + CD group, MPTP + CD group, and MPTP + MCT-KD group. Data are presented as the mean ± SEM. **p < 0.01, *p < 0.05 vs. the Ctrl group. ^##^*p* < 0.01, ^#^*p* < 0.05 vs. the MPTP + MCT-KD group. **C** Sample rank abundance curve for each group. **D** Boxplot of weighted Unifrac rank (*R* = 0.301, *p* = 0.001). **E** PCoA diagram based on the OTU matrix of mice fecal microbiota. **F** Heatmap analysis of the relative abundances of fecal microbiota at the family level in the three groups. **G** Relative abundances of fecal microbiota at the family level in the three groups. **H** Relative abundance of significantly altered fecal microbiota between the MPTP group and MPTP + MCT-KD group. Data are presented as the mean ± SEM. **p < 0.01, *p < 0.05 vs. the MPTP + MCT-KD group. **I** Mean Decrease Gini analysis of fecal microbiota alterations at the family level. **J** Graphical phylogenetic analysis of gut microbiota alterations among the three groups. Each dot represents the relative abundance of fecal microbiota. **K** Ternary phase diagram shows the ratio relationship of different fecal microbiota of the three groups. The circle sizes represent the average relative abundance of species. Statistical significance was determined using one-way ANOVA and Tukey’s tests for post hoc comparisons.

Because the gut microbiota plays an important role in PD development [44], we collected fecal samples from different groups of mice for 16 S RNA sequencing analysis in the fifth week. MPTP changed the gut microbiota structure of mice, as detected by α-diversity analysis and β-diversity analysis based on weighted UniFrac distances (Fig. 5A, B). Compared to the MPTP + CD group, the Shannon and Simpson indices were decreased in the MPTP + MCT-KD group. Figure 5C shows the rank abundance curve among three groups. There were no significant differences in weighted UniFrac rank between the Ctrl + CD, MPTP + CD, and MPTP + MCT-KD groups (Fig. 5D). To further identify the bacteria that play a key role in the MCT-KD for treating PD, we compared the relative abundance of microorganisms at different taxonomic levels between groups (Fig. 5E). The PCoA diagram shows the change in the gut microbiota structure in the PD model mice fed an MCT-KD. The changes in genus levels were also identified (Fig. 5F–H). Compared to the MPTP + CD group, the MPTP + MCT-KD group showed a decreased relative abundance of Aminobacterium, Desulfomicrobium, Fermentimonas, Fibrobacter, and Ruminiclostridium and an increased relative abundance of Blautia, Mycobacterium, and Ruminiclostridium. Figure 5I shows the mean decrease in the Gini coefficient of gut microbiota at the family level. Collectively, differential abundances of gut microbiota at various taxon levels among the three groups are summarized in Fig. 5J. Ternary phase diagram showed the changes in different groups of gut microbiota at the family level (Fig. 5K).

### MCT-KD treatment changed the fecal pellet metabolites in MPTP-treated mice

**Fig. 6:**
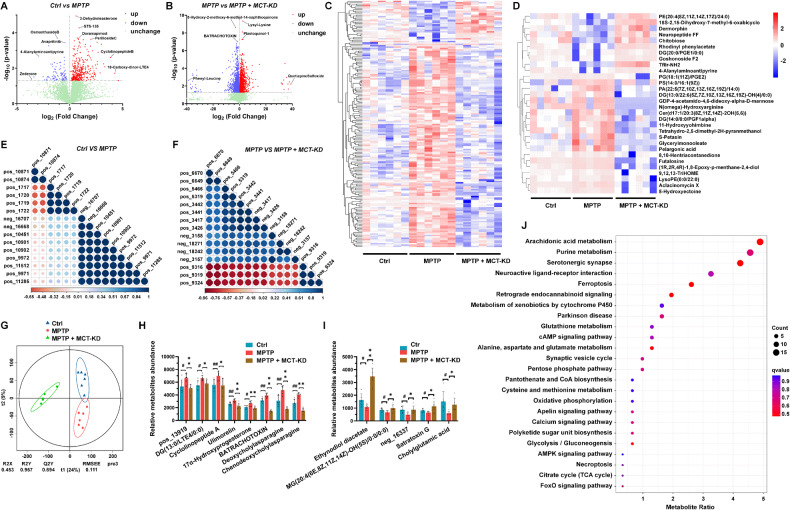
Metabolomic analysis of fecal pellets of MPTP-treated mice. **A**, **B** Volcano plots showing the DEGs between the Ctrl + CD group and MPTP + CD group, as well as between the MPTP + CD group and MPTP + MCT-KD group. **C**, **D** Hierarchical clustering of representative differential metabolites in fecal pellets among the three groups. **E** Correlation analysis of the top 15 different fecal pellet metabolites between the Ctrl group and MPTP group. **F** Correlation analysis of the top 15 different fecal pellet metabolites between the MPTP group and MPTP + MCT-KD group. **G** OPLS-DA analysis of fecal pellet metabolites among the three groups. **H**, **I** Relative abundance of the main differential fecal pellet metabolites among the three groups. **J** Differential metabolites in feces were analyzed by KEGG functional annotation and enrichment analysis. Data are presented as the mean ± SEM. **p < 0.01, *p < 0.05 vs. the MPTP + MCT-KD group. ^##^*p* < 0.01, ^#^*p* < 0.05 vs. the Ctrl group. Statistical significance was determined using one-way ANOVA and Tukey’s tests for post hoc comparisons.

Microbial metabolites may be the main mediators in factors involved in regulating the microbiota-gut-brain axis. To test this hypothesis, feces from mice in different treatment groups were sequenced by non-targeted metabolomics. Orthogonal projections to latent structures-discriminant analysis showed that the components of fecal metabolites changed significantly after 5 weeks of MCT-KD treatment compared to the MPTP group (Fig. 6G). The volcano map shows the overall trend of the difference in the metabolite content between the Ctrl + CD and MPTP + CD groups and between the MPTP + CD and MPTP + MCT-KD groups (Fig. 6A, B). We next clustered the differential metabolites and screened out 30 main differential metabolites (Fig. 6C, D). To determine the relationship between differential metabolites and PD mice, we conducted a correlation analysis of top 15 metabolites (Fig. 6E, F). The dose effect relationship of 13 differential metabolites is shown in Fig. 6H, I. Metabolic pathway analysis showed that the most relevant pathways related to metabolites in the MPTP group and the MPTP-KD group included arachidonic acid metabolism, purine metabolism, serotonergic synapse, neuroactive live receptor interaction, and ferroptosis (Fig. 6J). A complete list of main fecal pellet differential metabolites is given in sFig. 2. Taken together, these results indicate that MPTP can induce gut microbiota disorders and metabolic disorders in mice. We also analyzed the correlation between the above fecal metabolites and all bacterial genera (sFig. 3). Most of the differentiated metabolites were associated with microbiota abundance.

### MCT-KD treatment changed the profile of SNpc metabolites in mice

**Fig. 7:**
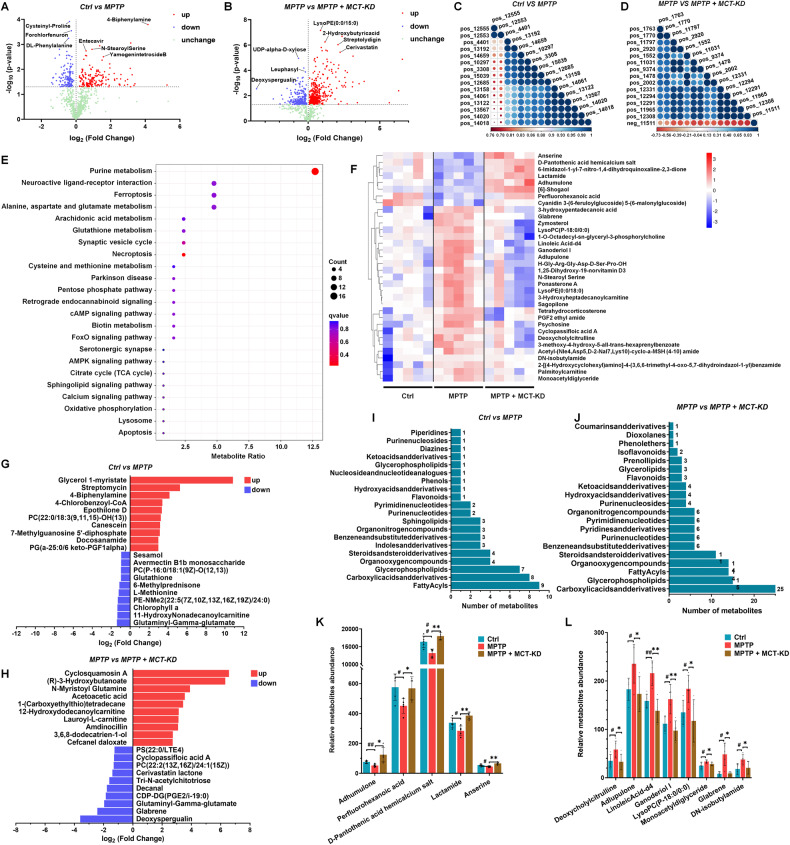
Metabolomic analysis of ketogenic diet treatment in the SNpc of MPTP-treated mice. **A**, **B** Volcano plots showing the DEGs between the Ctrl + CD group and MPTP + CD group, as well as between the MPTP + CD group and MPTP + MCT-KD group. **C** Correlation analysis of the top 15 different metabolites in the SNpc between the Ctrl + CD group and MPTP + CD group. **D** Correlation analysis of the top 15 different metabolites in SNpc between the MPTP + CD group and MPTP + MCT-KD group. **E** Functional annotation and enrichment analysis of differential metabolite KEGG. **F** Hierarchical clustering of representative differential metabolites in SNpc among three groups. **G** Functional annotation and enrichment analysis of differential metabolite KEGG between the Ctrl + CD group and MPTP + CD group. **H** Functional annotation and enrichment analysis of differential metabolite KEGG between the MPTP + CD group and MPTP + MCT-KD group. **I** Number of differential metabolites between the Ctrl + CD group and MPTP + CD group based on the HMDB database. **J** Number of differential metabolites between the MPTP + CD group and MPTP + MCT-KD group based on the HMDB database. **K**, **L** Relative differential metabolite abundance among three groups. Data are presented as the mean ± SEM. **p < 0.01, *p < 0.05 vs. the MPTP + MCT-KD group. ^##^*p* < 0.01, ^#^*p* < 0.05 vs. the Ctrl group. Statistical significance was determined using one-way ANOVA and Tukey’s tests for post hoc comparisons.

Dietary changes not only alter the structure of the gut microbiota but also change the metabolic phenotype of the host. To investigate whether MCT-KD affects the metabolism of the substantia nigra, the substantia nigra was isolated from mice after 5 weeks of MCT-KD treatment. The volcano map shows the differences in metabolites in the different groups (Fig. 7A, B). To further clarify the role of differential metabolites, we carried out correlation analysis and cluster analysis on differential metabolites and screened 33 main differential metabolites (Fig. 7C, D, F). We also used KEGG pairs for functional annotation and enrichment analysis (Fig. 7E). The functions of these differential metabolites mainly involve purine metabolism, neuroactive live receptor interaction, and ferroptosis. Fig. 7G, H shows the variation of fold differences between different groups of SN metabolites. The HMDB was used to classify and count the top 20 differential metabolites (Fig. 7I, J). Compared to the MPTP group, the changes in SN metabolite classification after long-term MCT-KD treatment were mainly associated with carboxylic acids and derivatives, glycosphospholipids, fatty acyls, organo-oxygen compounds, steroids, and steroid derivatives. Compared to the MPTP group, the metabolite abundance of anserine, perfluorohexanoic acid, D-pantothenic acid chemical salt, lactamide, and anserine increased to normal levels after MCT-KD treatment. However, the metabolite abundances of deoxycholylcitrulline, adlupulone, linoleicacid-d4, ganoderiol I, lysoPC (*p*-18:0/0:0), and glabrene decreased to normal levels after MCT-KD treatment (Fig. 7K, L). To expand and validate the transcriptomic findings, we performed correlation analysis of DEGs and differential metabolites in the mouse midbrain (sFig. 4).

### MCT-KD inhibited SN inflammation and reduced pro-inflammatory signals

**Fig. 8:**
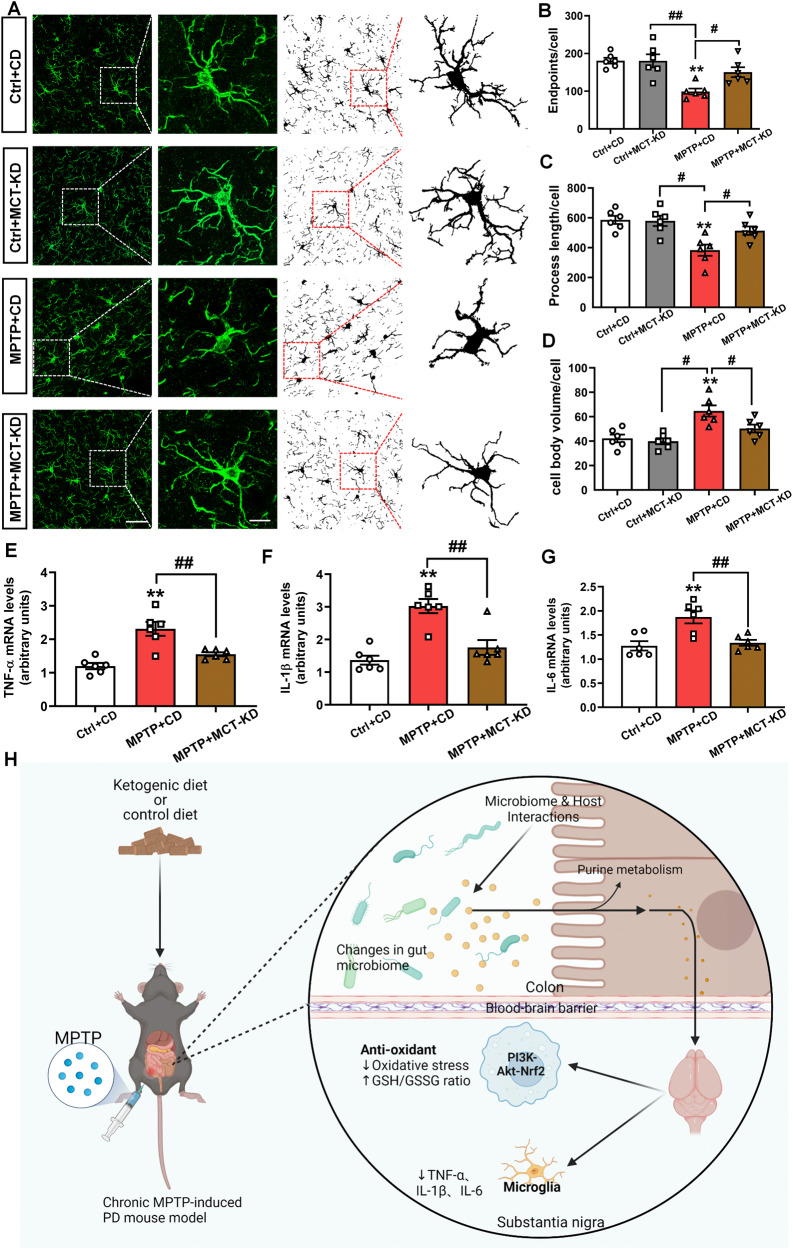
Ketogenic diet attenuates neuroinflammation in the SN of PD mice. **A** Iba1 was used to stain microglia in the Ctrl + CD group, Ctrl + MCT-KD group, MPTP + CD group, and MPTP + MCT-KD group. Maximum intensity projection of fluorescence images was transformed into binary images and then skeletonized. **B**–**D** Quantitative analysis of the number of endpoints, process length, and cell body volume of Iba1-positive cells after skeletonization; *n* = 6 per group. **E**–**G** Quantitative analysis of TNF-α, IL-1β, and IL-6 mRNA expression in the SN; *n* = 6 per group. Data are presented as the mean ± SEM. **p < 0.01 vs. the Ctrl + CD group. ^##^*p* < 0.01, ^#^*p* < 0.05 vs. the MPTP + CD group. Statistical significance was determined by one-way ANOVA and Tukey’s tests for post hoc comparisons. (**H**) Graphical abstract of the role of the ketogenic diet in Parkinson’s disease.

To further evaluate the effects of the MCT-KD on neuroinflammation, we used the microglia-specific marker Iba1 to detect the activation of microglia in the substantia nigra (Fig. 8A). The microglia in the Ctrl + CD group showed a characteristic static phenotype with fine-branching morphology. The MPTP + CD group showed more Iba1 + cells, which changed to the shape of large and round phagocytes. Strikingly, the MCT-KD reversed the morphological changes in microglia induced by MPTP. Compared to the Ctrl + CD group, the microglia endpoint and process length decreased and the cell body volume increased in the MPTP + CD group. These changes were reversed by the MCT-KD (Fig. 8B–D). To further explore the inflammatory state in the brain, we measured the mRNA levels of TNF-α, IL-1β, and IL-6 in SNpc by qPCR (Fig. 8E–G). Consistent with the expected results, MPTP increased the expression level of TNF-α, IL-1β, and IL-6, whereas the MCT-KD decreased the expression of these proinflammatory factors.

### Discussion

Our data showed that the MCT-KD can rescue DA neurons death and motor dysfunction in MPTP-induced PD mice model. The mechanism may be that MCT-KD treatment increased ketone BHB and reduced blood glucose levels, inhibited oxidative stress by enhancing the PI3k/Akt/Nrf2 pathway, increased the expression of GSH and SOD, and inhibited the activation of microglia. The MCT-KD also rescue mitochondrial loss and increased ATP production in MPTP-induced PD mice model. Additionally, MCT-KD altered the gut microbiota and metabolism of substantia nigra may through changing gut microbiota metabolites in which involving purine metabolism. We also demonstrated that BHB increased superoxide dismutase (SOD) activity, GSH concentration and GSH / glutathione disulfide (GSSG) ratio in vitro. Additionally, BHB restored the reduced state of NADPH and its oxidized state (NADP^+^) ratio. These results suggest that MCT-KD prevent DA loss by increasing BHB.

Mitochondrial damage and ROS accumulation leads to DA neuron loss, it was believed that KD conferred neuroprotection through its potential as a substrate for energy substitution. Studies have shown that the activated PI3K/Akt signaling pathway is crucial for protecting neurons from oxidative stress [45], Nrf2 is a key downstream element of the PI3K/Akt signaling pathway, which is involved in the transduction of various signals from the cell surface to the nucleus. When the PI3K/Akt pathway is activated, Akt phosphorylates Nrf2, which is dissociated from Keap1 and transferred to the nucleus to regulate the expression of antioxidant proteins such as HO-1 and NQO1 through interaction with ARE [46]. It has suggested that KD can increase the expression of Nrf2 and in conformance with our result [47]. MCT-KD also increased the expression of SOD and GSH, and BHB can directly enhance the antioxidant stress capacity of DA neurons. Also, MCT-KD administration reduced mitochondrial loss in PD and promoted the production of ATP, thus rescuing the reduction of oxidative respiratory chain ATP in DA neurons caused by MPTP. These results suggest that the neuroprotective effect of MCT-KD is partially attributed to antioxidant properties of DA neurons by the PI3K/Akt/Nrf2 pathway and reducing mitochondrial loss, promoting ATP production.

It has been reported that gut microbiota imbalances can affect the central nervous system and intestinal nervous system [44]. Also, it has shown that an abnormal microbial community structure existed in MPTP-induced PD mice [48–55]. The abundance of several short-chain fatty acid (SCFA)–producing bacteria, including Blautia and Romboutsia, is decreased in PD [56, 57]. We detected the intestinal microbial composition of chronic PD mice and the effect of MCT-KD on the gut microbiota of mice by 16sRNA-sequencing analysis. Compared to the MPTP + CD group, the relative abundance of Blautia, Mycobacterium, and Romboutsia was significantly increased in the MPTP + MCT-KD group, while the relative abundance of Desulfomicrobium was significantly decreased. The low abundance of SCFA-producing bacteria may affect the integrity of the intestinal barrier and immune function in patients with PD [58]. Collectively, our data suggest that MCT-KD may protect MPTP-induced PD mice by reconstituting normal microbial communities.

To further explore the protective mechanism of MCT-KD treatment in PD, we introduced non-targeted metabolomics. The results showed that the differential metabolites produced by the fecal flora of mice were mainly involved in arachidonic acid metabolism and purine metabolism, while the differential metabolites in the midbrain were mainly involved in purine metabolism. To some extent, this indicates that MCT-KD may exert a therapeutic effect by regulating purine metabolism. Purines are heterocyclic aromatic organic compounds that constitute the core of DNA, RNA, nucleosides, and nucleotides. Nucleotides are involved in many important metabolic pathways, including cell signaling and energy metabolism. Adenosine receptor signaling also regulates the permeability of the blood-brain barrier [59]. Studies have shown that purine metabolism is altered in PD, and oxidative damage to nucleic acids has been found in the PD brain [60–62]. Interestingly, in our study, MPTP did not affect the metabolic pathway of purine in the mouse feces and midbrain. The MCT-KD downregulated most purine metabolites in the feces of mice, including IMP, deoxyinosine, and hypoxanthine, and affected purine metabolism, including IDP and deoxyinosine, in the SNpc of mice. Compared to the MPTP group, the gene expression related to purine metabolism in the MPTP + MCT-KD group did not change significantly, and the mRNA expression of *Nme*3 decreased, indicating that an MCT-KD may improve the metabolic disorder in the brains of PD mice by altering the metabolites of gut microbiota. However, the specific mechanism still needs further study and explanation.

In this study, we confirmed that a long-term MCT-KD can protect against MPTP-induced DA neuron loss and altered the gut microbiota and its metabolites in mice. This study also highlights the effects of dietary changes on the gut-brain axis. Although the related problems of ketogenic diet in purine metabolism still need to be solved, our study provides more evidence for the potential neuroprotective effect of a ketogenic diet in PD.

## Materials and methods

### Antibodies, reagents, and animals

MPTP was purchased from Sigma-Aldrich (St. Louis, MO, USA); anti-TH (F-11, sc-25269) and dopamine transporter (DAT, sc-32258) antibodies were purchased from Santa Cruz Biotechnology (Dallas, TX, USA); anti-BDNF (ab108319), SHDA (ab137040), and SDHB (ab178423) antibodies were purchased from Abcam (Cambridge, MA, USA); anti-Bax (#14796), Phospho-PI3 Kinase (#17366), PI3 K (#4249), Phospho-Akt (#4060), and Akt (#4685) antibodies were purchased from Cell Signaling Technology (Danvers, MA, USA); Nrf2 (16396-1-AP), Bcl-2 (16396-1-AP), and GAPDH (60004–1) antibodies were purchased from the Proteintech Group (Rosemont, IL, USA); DyLight 488 goat anti-mouse IgG (H + L) (70-GAM4882) and DyLight 594 goat anti-rabbit IgG (H + L) (70-GAR5942) were purchased from Multi Sciences (Hangzhou, China); horseradish peroxidase (HRP)-labeled goat anti-rabbit IgG and HRP-labeled goat anti-mouse IgG were purchased from Beyotime Biotechnology (Shanghai, China); and the GSH and GSSG Assay Kit (#S0053) and Cu/Zn-SOD and Mn-SOD Assay Kit with WST-8 (#S0103) were purchased from Beyotime Biotechnology (Shanghai, China).

#### Animals

Male C57BL/6 mice (*n* = 40), aged 10 weeks (weight, 25 ± 2 g), were obtained from Zhuhai BesTest Bio-Tech Co., Ltd. (Zhuhai, China). Calculations for sample sizes were performed using an online sample size calculator (https://clincalc.com/stats/samplesize.aspx). The allocation of mice in each group were randomized and blinded. The mice were housed in an ambient temperature environment (22 ± 2 °C) under a 12-h dark/light cycle ((07:00, lights on; 19:00, lights off), with free access to water and food). All animal experiments were performed according to the Experimental Animal Ethics Committee of Zhujiang Hospital of Southern Medical University (approval number: LAEC-2021-132).

#### PD mouse model induction and ketone diet treatment

MPTP (25 mg/kg) was administered intraperitoneally continuously twice a week over a 5-weeks period to induce the chronic PD model.

Mice were divided into four groups (*n* = 10 per group): (i) control mice fed a CD (Ctrl + CD); (ii) MPTP-treated control mice fed a CD (MPTP + CD); (iii) control mice fed an MCT-KD (Ctrl + MCT-KD); and (iv) MPTP-treated mice fed an MCT-KD (MPTP + MCT-KD). The treatment animals received an MCT-KD (16.6% protein, 0% carbohydrates, 66.5% fat [mainly composed of coconut oil], with respect to chow kcal%), while control animals received a CD (10.0% protein, 79.9% carbohydrates, 10.0% fat [mainly composed of coconut oil], with respect to chow kcal%). The per calorie macronutrient content for the diets were customized based on the formula used in a previous work [63] and were purchased from Jiangsu Xietong Pharmaceutical Bioengineering Co., Ltd. (Jiangyin, China). Table 1 provides the detailed composition of the feeds used in this study. Mice were fed an MCT-KD 1 week in advance to induce hyperketonemia before the intraperitoneal administration of MPTP.

**Table 1:** Composition and energy of the control diet and MCT-KD.

Macronutrients	Control diet	MCT-KD
Protein, kcal%	9.99%	9.99%
Carbohydrates, kcal%	79.94%	0.00%
Fat, kcal%	9.97%	89.91%
Energy, kcal/gm	3.8	6.7
Ingredient	gm	gm
Casein	100	100
L-Cysteine	1.5	1.5
Corn starch	371	0
Maltodextrin	35	0
Sucrose	406	0
Cellulose	50	50
Soybean oil	25	25
Coconut oil	20	381
Mineral mix, S10026B	50	50
Vitamin mix, V10001C, 10 × Vits	1	1
Choline bitartrate	2	2

### Behavior tests

#### Open field test

This method was modified from a reported protocol [64]. Before the experiment, mice were placed into the behavioral laboratory for adaptation for >6 h. In the open field experiment, the mice were gently placed in the experimental box (40 cm × 40 cm × 40 cm) at the center. The movement track of the mice was recorded with a camera, and the experiment was timed for 15 min. Noldus Etho Vision XT software was used to analyze the total distance moved and the average speed of the mice in the experimental box.

#### Pole test

This method was carried out as described in our previous work [65]. Mice were pre-trained with the pole (height, 75 cm; diameter, 9 mm; wrapped with gauze) three times to ensure that all mice would climb down smoothly when they were placed head up on the top of a pole. The total time it took the mice to climb down the pole was recorded.

#### Rotarod test

The rotarod test was implemented as described previously [66]. Mice were placed on a Rotarod (Panlab LE8505, USA) and were trained at 10 rpm for 3 days. During the test, the rotarod speed was increased from 4 to 30 rpm in 300 s, and the time to fall was recorded.

#### Grasping test

The muscle grip strength was assessed using the grasping test as described previously [67]. Mice were allowed to use their front paws to grasp a horizontal wire (diameter, 1 mm; 35 cm from the ground) for 10 s. During this time, the grasping score was recorded as follows: grasping the wire with both hind legs achieved a score of 3; grasping the wire with only one hind leg achieved a score of 2; grasping the wire with either hind leg achieved a score of 1; and dropping achieved a score of 0. The final score was determined by the average score of the three trials.

### Cell culture and drug treatments

MN9D cells were purchased from American Type Culture Collection (ATCC, Manassas, VA, USA) and were cultured in Dulbecco’s Modified Eagle Medium (GIBCO, Carlsbad, CA, USA) containing 8% fetal bovine serum (GIBCO, Carlsbad, CA, USA), 2 U mL^−1^ penicillin (Beyotime Biotechnology), and 2 mg mL^−1^ streptomycin (Beyotime Biotechnology) at 37 °C in a humidified atmosphere containing 5% CO_2_. The medium was changed every 2 days. A MN9D cell suspension (≈5 × 10^4^ cells) was added to 24-well plates for 24 h.To investigate the effects of BHB, we pretreated MN9D cells with 4 mM BHB prior to 1 mM MPP+ for 4 h [68].

### Blood glucose and blood ketone body detection

The tail vein of the mouse was dropped on the corresponding test paper. The blood glucose test was conducted with ~2 µL of blood, and the blood ketone body test was conducted with ~5 µL of blood. A Bayer blood glucose meter was used to detect blood glucose, and an Abbott blood glucose ketone meter was used to detect the ketone body.

### Determination of the neurotransmitter and metabolite levels

The levels of dopamine, 3,4-dihydroxyphenylacetic acid (DOPAC), serotonin (5-HT), and 5-hydroxyindoleacetic acid (5-HIAA) were assessed by HPLC-MS/MS. First, a certain number of tissue samples were accurately weighed and their weights were recorded. Next, 250 µL methanol (containing 0.1% formic acid) was added and vortexed for 1 min, before homogenizing for 3 min. The supernatant was obtained by high-speed centrifugation at 14000 rpm for 10 min. The samples were then subjected to HPLC-MS/MS analysis. LC-MS/MS analysis was performed using UltiMate 3000 RS (Thermo Fisher) and Q Exactive (Thermo Fisher). The Q Exactive series mass spectrometer operates in positive/negative mode with the following settings: spray voltage, 3.2 kV; capillary temperature, 300 °C; collision gas, high purity argon (purity ≥99.999%); sheath gas, nitrogen, 40 arb; auxiliary gas (Aux gas heater temp) nitrogen, 350 °C. The samples were injected into a Waters T3 column (150 mm × 2.1 mm, 3 µm) at a flow rate of 0.3 mL/min. The eluent in the aqueous phase mode was an aqueous solution of 0.1% formic acid, and the eluent in the organic phase was 0.1% ethyl formate. Chromatogram acquisition and integration of analytes were processed by Xcalibur 4.0 software (Thermo Fisher). Taking the peak area as the ordinate and the concentration as the abscissa, the standard curve was obtained via weighted coefficient regression. The content of each sample was obtained by regression analysis.

### Immunohistochemistry and immunofluorescence assays

Embedded mouse brains were cut into 15-µm sections with a freezing microtome (Leica), and the slices were incubated with corresponding primary antibodies overnight at 4 °C. For the immunohistochemistry assay, brain slices were incubated with a secondary antibody labeled with biotin and stained with diaminobenzidine (DAB). The images were scanned under a microscope (Leica CS2, Hamburg, Germany). For the immunofluorescent assay, brain slices were incubated with fluorescent‐labeled secondary antibody, and DAPI was used to stain nuclei. Images were scanned under a confocal laser‐scanning microscope (SP8; Leica). Quantitative analysis was performed using the Image‐Pro Plus 6.0 phonogram analysis system (IPP 6.0, Media Cybernetics, Bethesda, MD, USA).

### Transmission electron microscope

The ultrastructural morphology of the mitochondria in substantia nigra was analyzed using a transmission electron microscope (TEM). Fixed substantia nigra tissues were dehydrated with an increasing gradient of alcohol and acetone and then embedded with 812 embedding agent (SPI‐Pon 812 Epoxy Resin Monomer; SPI, Shanxi, China). The sliced sections were double‐stained with uranium lead and dried overnight at RT, before imaging and analyzing by transmission electron microscopy (HT7700; Hitachi, Tokyo, Japan).

### Western blotting

Substantia nigra (SN) and striatum tissues were collected, and total protein was extracted with RIPA Lysis Buffer (Beyotime Biotechnology) and quantified using a Bicinchoninic Acid (BCA) Protein Assay Kit (Beyotime Biotechnology). The samples were separated by SDS‐PAGE and transferred to PVDF membranes. The membranes were then blocked with 5% bovine serum albumin (BSA) and incubated with primary antibody. After incubation with secondary antibody, chemiluminescence was visualized on the GeneGnome XRQ Chemiluminescence Imaging System (Gene Company, Hong Kong, China). Image J software was used to analyze the optical density of the bands.

### RNA-seq

Total RNA was extracted from the SN tissue using TRIzol (Life Technologies, Carlsbad, CA, USA). According to the manufacturer’s instructions, sequencing libraries were prepared using the NEBNext UltraTM RNA Library Prep Kit for Illumina (NEB, USA). Next, the PCR products were purified (AMPure XP system) and the library quality was assessed on an Agilent Bioanalyzer 2100 system. The clustering of the index-coded samples was performed on a cBot Cluster Generation System using a TruSeq PE Cluster Kit v4-cBot-HS (Illumina) according to the manufacturer’s instructions. After cluster generation, the library preparations were sequenced on an Illumina platform and paired-end reads were generated. The raw reads were further processed with a bioinformatic pipeline tool, BMKCloud (www.biocloud.net) online platform. The sequencing results were adjusted using the Benjamin Hochberg method to control the error detection rate. Gene Ontology (GO) enrichment analysis and Kyoto Encyclopedia of Genes and Genomes (KEGG) pathway enrichment analysis were used to analyze the results. RNAseq data used in this study are available under GEO: GSE232039.

### 16 S rDNA analysis of fecal samples

Total RNA was extracted from nigral samples using TRIzol (Life Technologies, Carlsbad, CA, USA). Next, RNA libraries were prepared using the NEBNext UltraTM RNA Library Prep Kit for Illumina (NEB, USA) according to the manufacturer’s instructions. The quality of the purified libraries was assessed using an Agilent Bioanalyzer 2100 system. Clustering of the index‐coded samples was performed on a cBot Cluster Generation System using the TruSeq PE Cluster Kit v3‐cBot‐HS (Illumina), according to the manufacturer’s instructions. After cluster generation, the samples were sequenced on an Illumina HiSeq 2500 platform (San Diego, CA, USA). HTSeq v0.6.0 was used to count the read numbers mapped to each gene, and the fragments per kilobase of transcript‐per‐million mapped reads (FPKM) of each gene were calculated based on the length of the gene and read counts mapped to the analyzed gene. Differentially expressed gene (DEG) analysis was performed using the DESeq2 R package (1.10.1). The resulting *p*‐values were adjusted using the Benjamini–Hochberg approach for controlling false discovery rates. Functional enrichment of DEGs was analyzed by the KEGG database.

### Metabolomics and analysis

The appropriate grinding and extraction methods were selected according to the sample type, and the metabolites were extracted into the extraction solution (methanol acetonitrile volume ratio = 1:1; internal standard concentration = 2 mg/L), before ultrasonicating for 10 min (ice water bath). Next, the sample was allowed to stand for 1 h at −20 °C, before centrifuging at 4 °C at 12,000 rpm for 15 min. Next, 500 μL of supernatant was transferred to an EP tube and dried in a vacuum concentrator. Subsequently, dried metabolites were added with 160 μL of extraction solution (acetonitrile water volume ratio: 1:1) to redissolve, and the mixture was vortexed for 30 s and immersed in an ice water bath for ultrasonification for 10 min. Next, the sample was centrifuged at 4 °C at 12,000 rpm for 15 min, before carefully removing 120 μL supernatant to a 2-mL injection bottle. Ten microliters of each sample were mixed with the QC sample for machine detection. The samples were tested on the Waters Acquity I-Class PLUS ultra-high performance liquid tandem Waters Xevo G2-XS QT of high-resolution mass spectrometer using a water acquisition UPLC HSS T3 column (1.8 μm, 2.1 × 100 mm). Metabolites were measured by Biomarker Technologies (Beijing, China). Metabolome data statistics were performed on a platform (Biomarker Technologies Corporation, China). The identified compounds were searched for classification and pathway information in the KEGG, Human Metabolome Database (HMDB), and lipidmaps databases. Principal component analysis and Spearman correlation analysis were used to judge the repeatability of samples in the group and quality control samples. According to the grouping information, the differential multiple was calculated and compared, and the difference significance *p*-value of each compound was calculated by *t*-test. The differential multiple, *p*-value, and VIP value of the orthogonal partial least squares discriminant analysis (OPLS-DA) model were combined to screen differential metabolites. The screening criteria were FC > *p*-value < 0.05 and VIP > 1. A hypergeometric distribution test was used to determine the differential metabolites with significant enrichment in the KEGG pathway.

### Mitochondrial superoxide detection assay

MitoSOX (ThermoFisher Scientific) and Hoechst 33342 Staining (Beyotime Biotechnology) working solutions were added to cells. After incubation for 10 min at 37 °C, the cells were washed three times. The images were scanned under a confocal laser-scanning microscope (SP8, Leica).

### ROS detection assay

Hoechst 33342 Staining and 2,7-dichlorofluorescein diacetate (Beyotime Biotechnology) working solutions were added to cells. After incubation for 20 min at 37 °C, the cells were washed three times. The images were scanned under a confocal laser-scanning microscope (SP8; Leica).

### SOD detection assay

A Cu/Zn-SOD Assay Kit with WST-8 (Beyotime Biotechnology) was used to determine the intracellular SOD activity. Briefly, SN tissues were homogenized and incubated with WST-8/enzyme working solution at 37 °C for 30 min. The absorbance was measured at a wavelength of 450 nm with a microplate reader (PerkinElmer). The protein concentrations were determined using the BCA assay, and the results were expressed as units per mg protein (U mg^−1^ protein).

### GSH and GSSG detection assay

Glutathione (GSH) and glutathione disulfide (GSSG) detection was performed using the GSH/GSSG kit (S0053, Beyotime Biotechnology) according to the manufacturer’s instructions. Tissues were collected and Protein Removal solution was added. The samples were then freeze-thawed twice rapidly in liquid nitrogen and 37 °C water alternatively. After centrifugation at 10,000 × g for 10 min, the supernatant was used for the determination of total glutathione. GSH-scavenging reagent working solution was added to degrade GSH. Then, total glutathione-working solution and NADPH solution were added to the supernatant to detect the concentration of total glutathione and GSSG. The GSH concentration was obtained from the total glutathione concentration minus double the GSSG concentration, and the GSH/GSSG ratio was determined. The absorbance at 412 nm was measured using a microplate reader (PerkinElmer). Data were obtained from three separate experiments, each of which was performed in triplicate.

### NADPH/NADP^+^ detection assay

The MN9D cellular NADPH/NADP^+^ ratio was detected using an NADP^+^/NADPH assay kit (S0179, Beyotime Biotechnology) according to the manufacturer’s instructions. The samples were lysed using NADP^+^/NADPH extract, followed by 12,000 g centrifugation at 4 °C for 10 min. Then, the supernatant was mixed with G6PDH working solution and incubated at 37 °C for 10 min. Next, 10 μl of chromogenic solution was added to the mixture, and the mixture was incubated at 37 °C for 20 min. The absorbance at 450 nm was measured using a microplate reader (PerkinElmer). Data were obtained from three separate experiments, each of which was performed in triplicate.

### ATP detection assay

ATP levels in SN were measured using an Enhanced ATP Assay Kit (Beyotime Biotechnology). Briefly, lysis buffer was added and the samples were ultrasonically homogenized. Lysates were then clarified via centrifugation (4 °C, 12,000 × g, 5 min), and the supernatants were collected and transferred to a new tube. Then, 20 μL of each supernatant was mixed with 100 μL ATP detection working dilution. Luminance was assayed in a chemiluminescence instrument (luminometer).

### Quantitative RT‐PCR

To perform RT-PCR, total RNA from substantia nigra tissues was extracted with Trizol, and genomic DNA was removed with DNA Eraser Buffer. RNA was then reverse transcribed into cDNA, and the qPCR was performed with TB Green Premix ExTaq (Takara). Forty cycles (95 °C for 5 s, 55 °C for 30 s, and 72 °C for 30 s) were set up for the reaction with the following primer sequences: TNF‐α, forward 5′‐ CACGTCGTAGCAAACCACC‐3′ and reverse 5′‐TGAGATCCATGCCGTTGGC‐3′; IL‐1β, forward 5′‐AATGCCACCTTTTGACAGTGAT‐3′ and reverse 5′‐ TGCTGCGAGATTTGAAGCTG‐3′; IL‐6, forward 5′‐AGGATACCACTCCCAACAGACC‐3′ and reverse 5′‐AAGTGCATCATCGTTCATACA‐3′.

### Statistical analysis

All data are presented as the mean ± standard error of the mean (SEM). Statistical analysis of multiple comparisons was performed by one-way analysis of variance (ANOVA), followed by Tukey’s post‐hoc test. Metabolomic analysis was conducted using the Hotelling T2 test. To obtain the correlations between different experiments, Spearman correlation analysis was conducted using GraphPad Prism 8.0. Differences with a *p*‐value < 0.05 were considered statistically significant.

## Supplementary information

Original western blots

Supplementary figure legends

Supplementary Fig. 1

Supplementary Fig. 2

Supplementary Fig. 3

Supplementary Fig. 4

Reproducibility Checklist

## Data Availability

The raw data of this article will be made available by the authors, without undue reservation.

## Appendix A. supporting information

Supplementary data relevant to this article can be found in the supplementary files.
