# Supplementary material for: Neuroprotective effect of a medium-chain triglyceride ketogenic diet on MPTP-induced Parkinson’s disease mice: a combination of transcriptomics and metabolomics in the substantia nigra and fecal microbiome
Source: Cell Death Discov. 2023 Jul 17;9:251. doi: 10.1038/s41420-023-01549-0 (PMC10352270; doi:10.1038/s41420-023-01549-0)
Supplement: Supplementary file 1 — Original western blots [file 41420_2023_1549_MOESM1_ESM.docx]

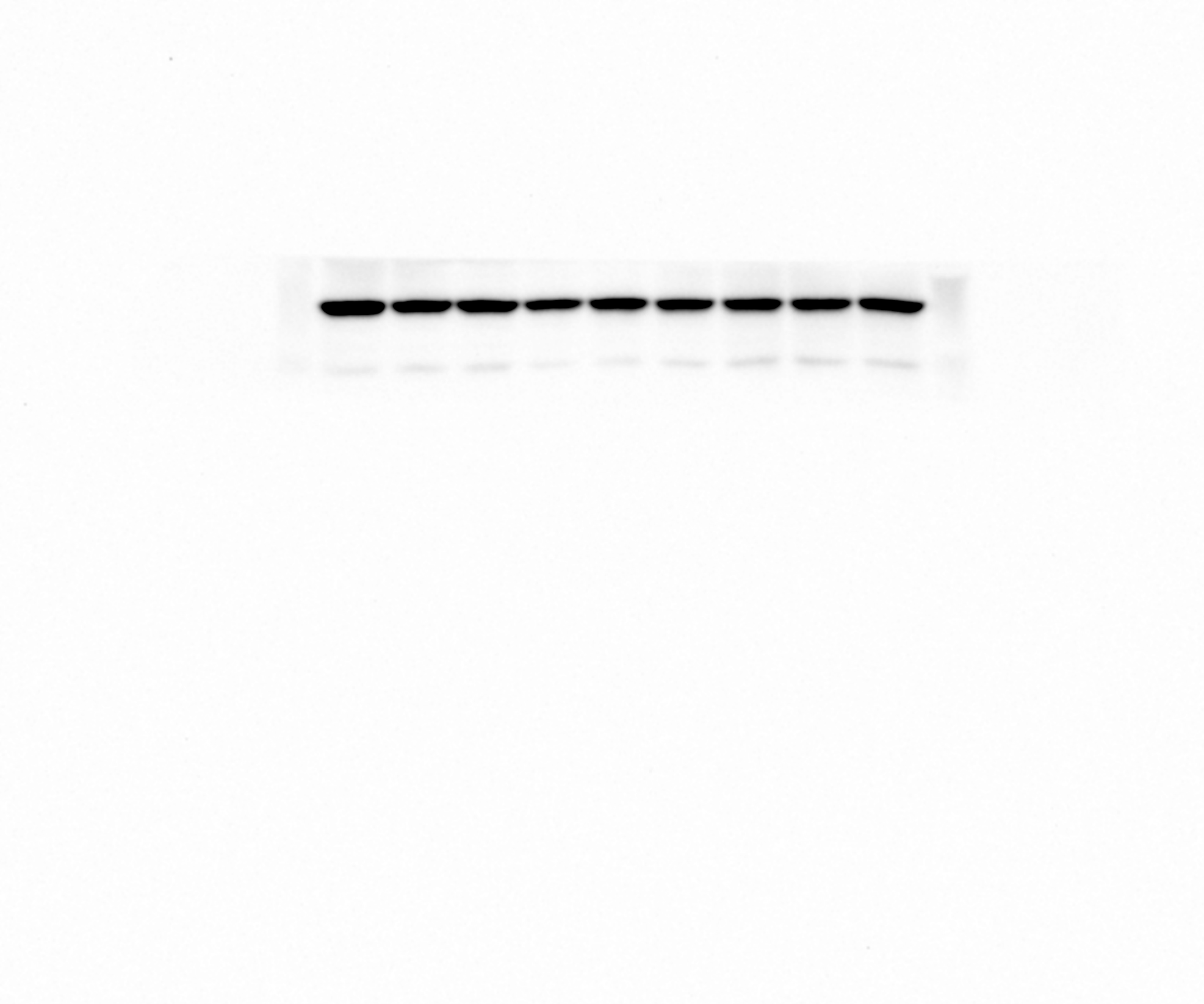

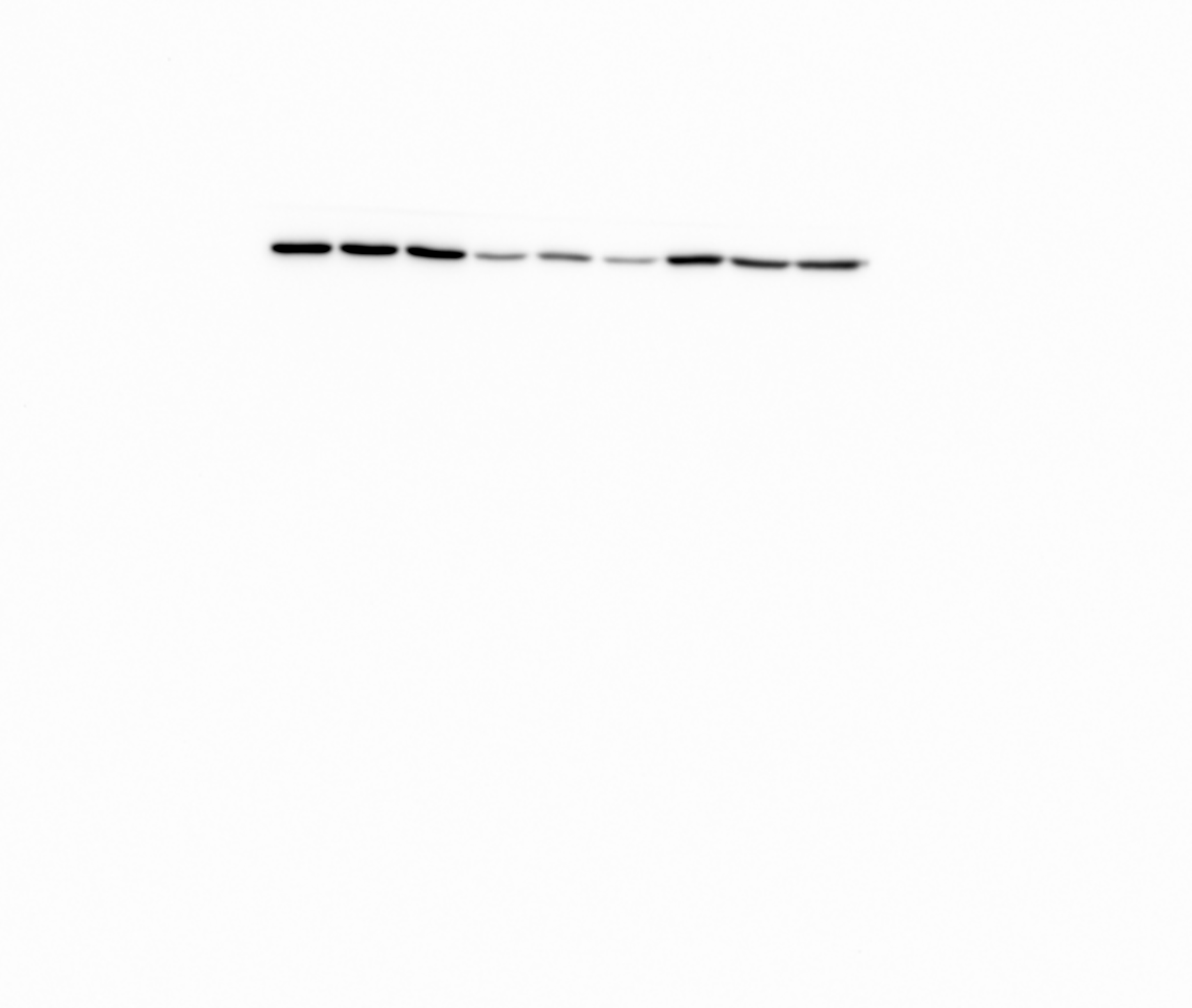

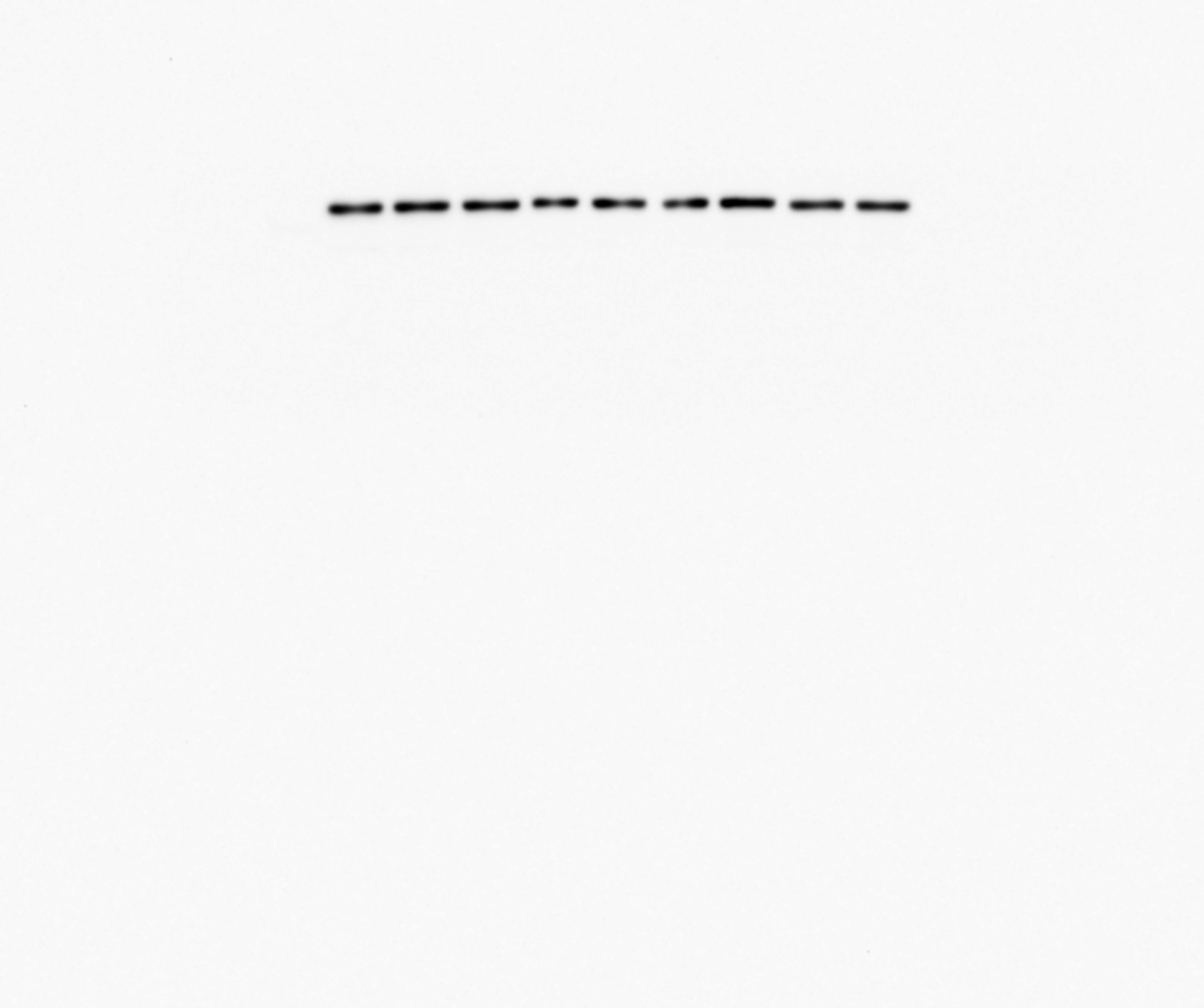


**Substantia nigra**

**~37 KD-**

**~80 KD-**

**Th**

**~60 KD-**

**C**

**Ctrl+CD MPTP+CD MPTP+KD**

**DAT**

**GAPDH**


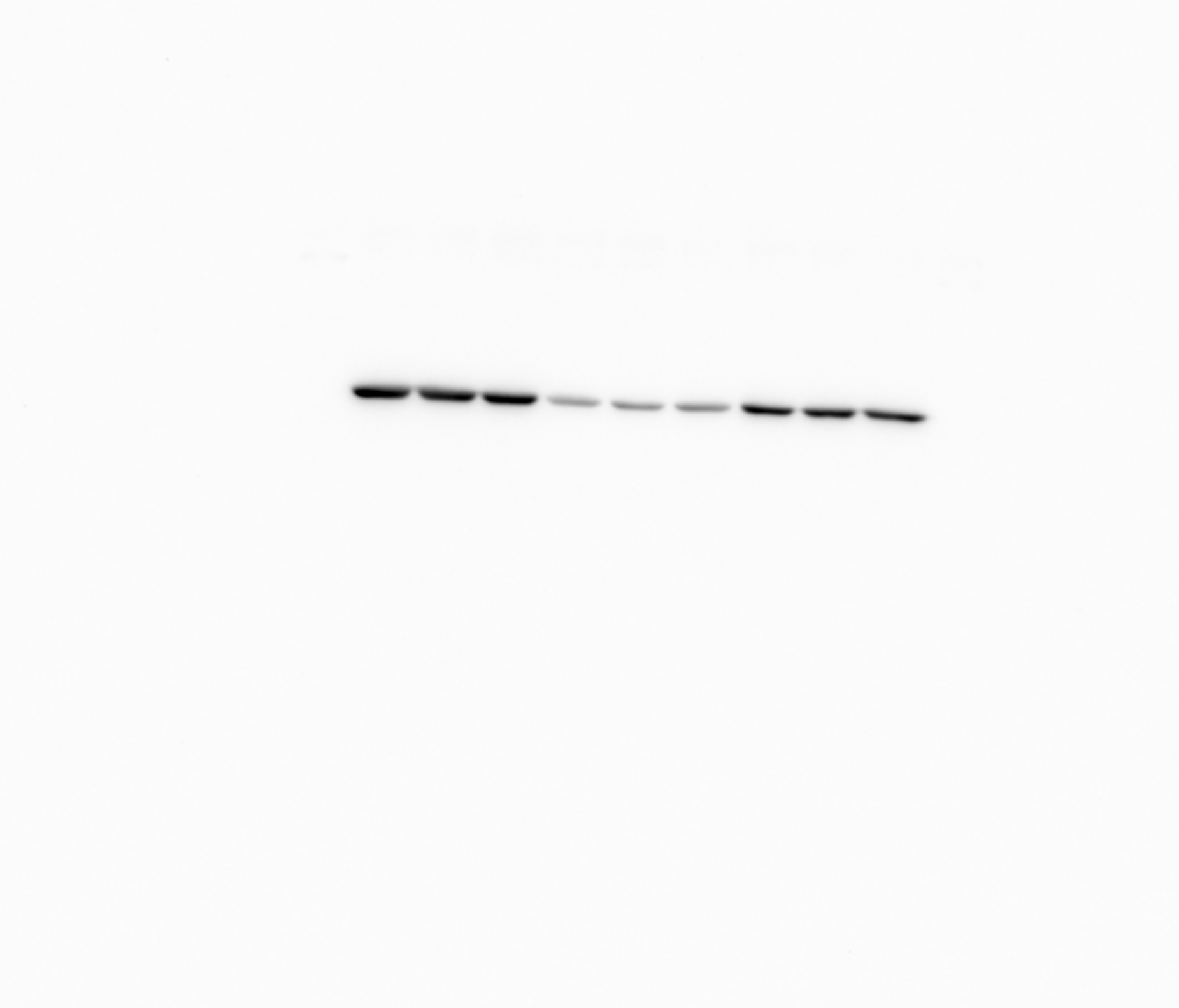

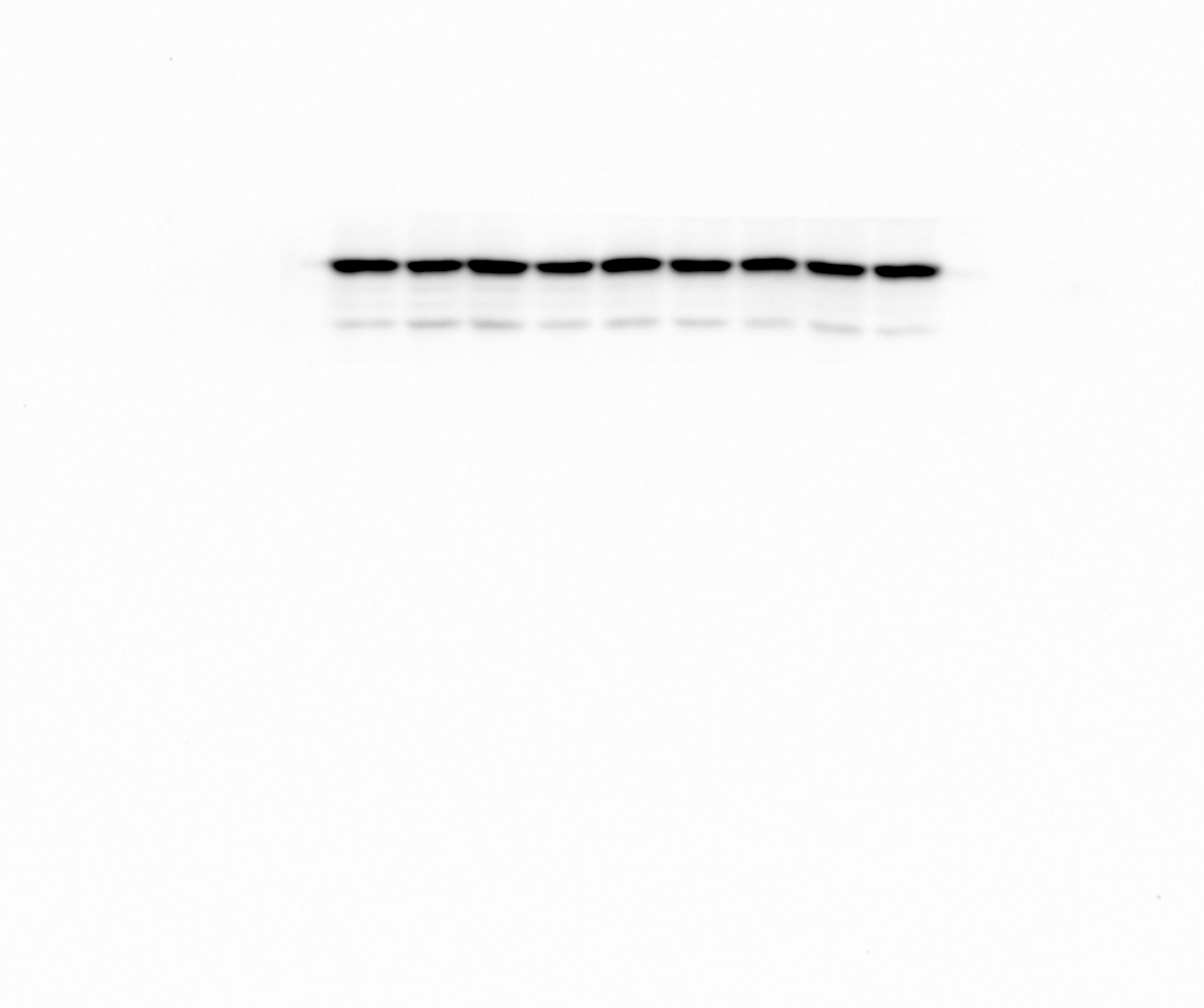


**D**

**GAPDH**

**~37 KD-**

**Striatum**

**Ctrl+CD MPTP+CD MPTP+KD**

**~60 KD-**

**Th**


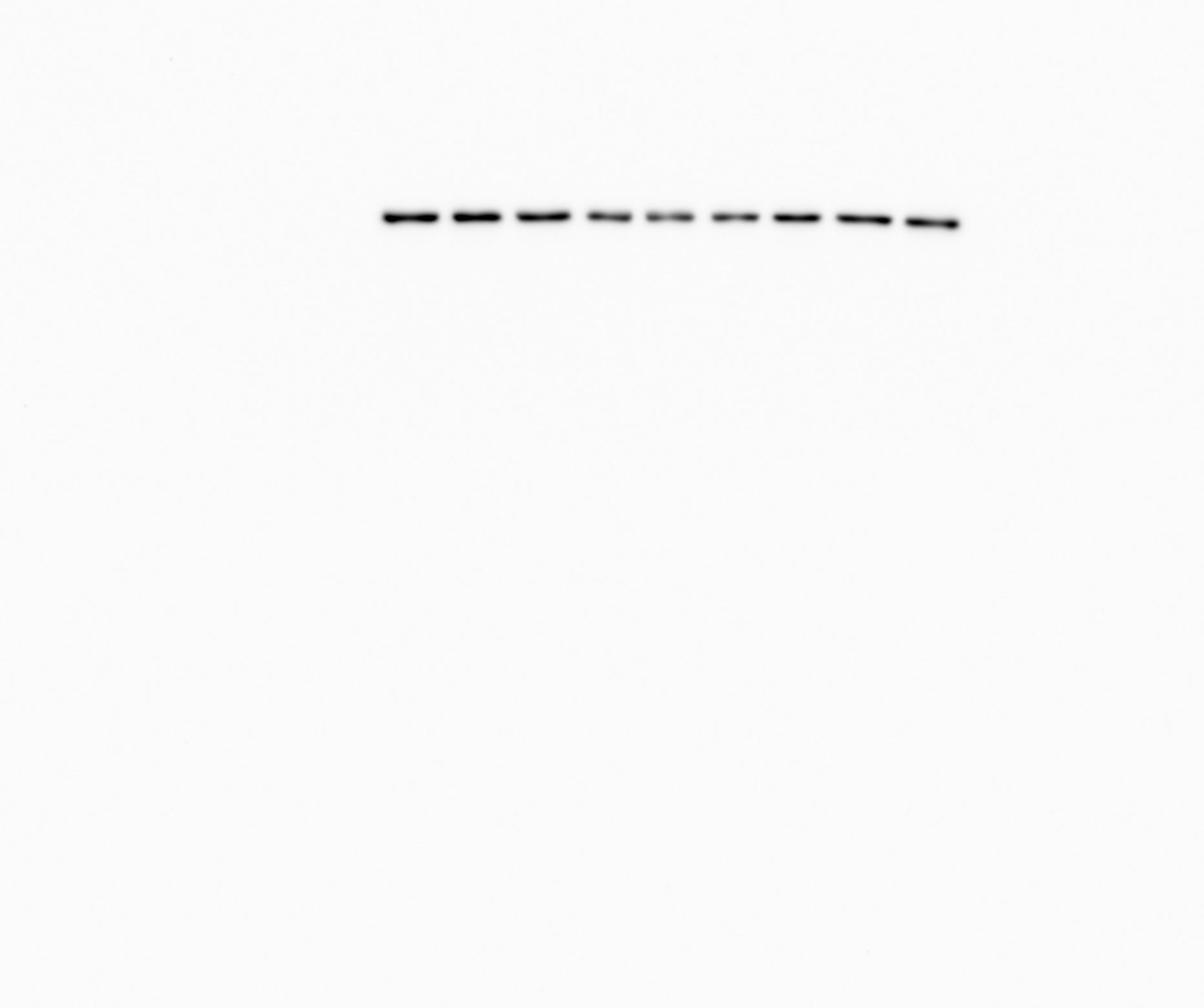


**~80 KD-**

**DAT**


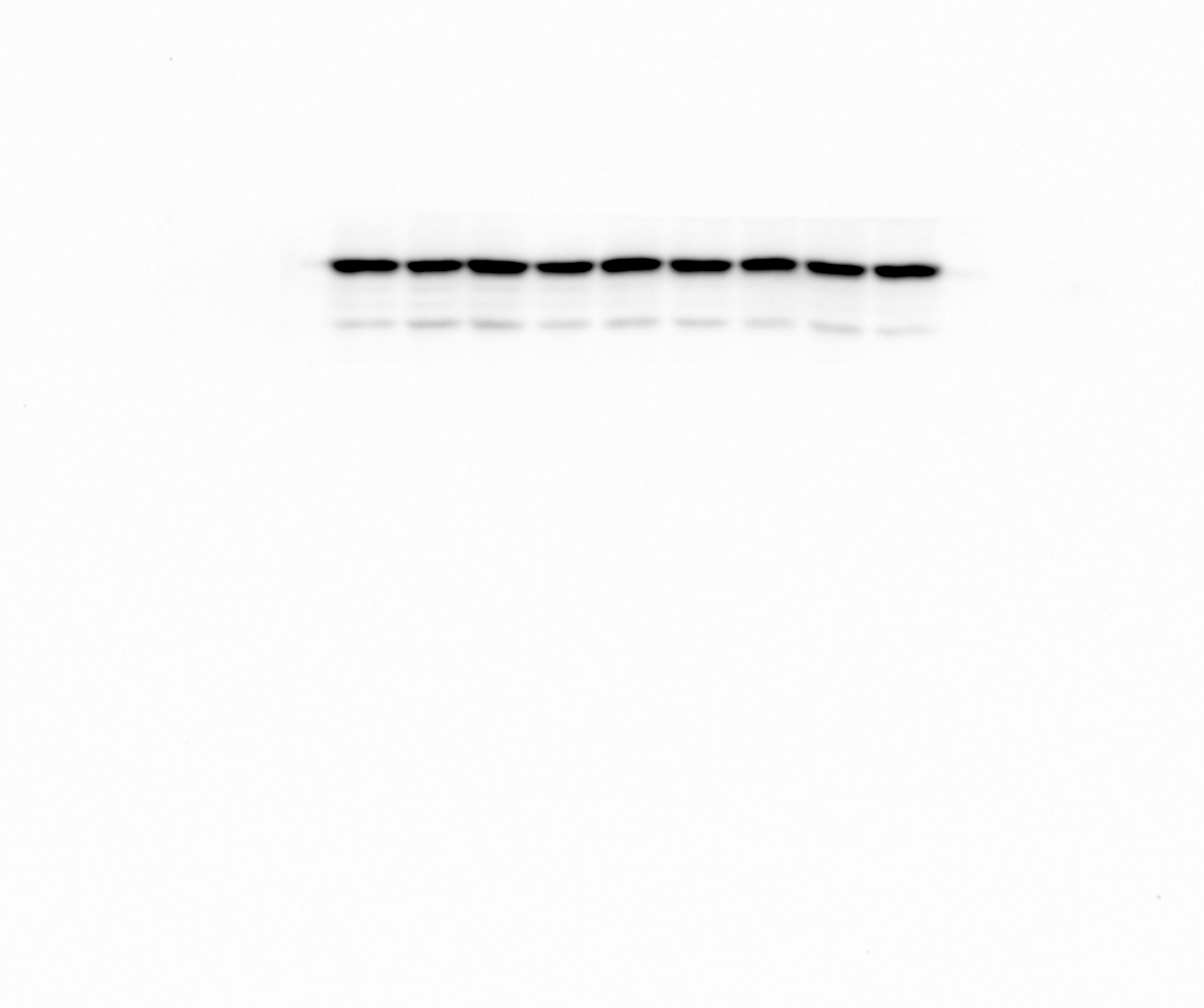


**GAPDH**

**~37 KD-**


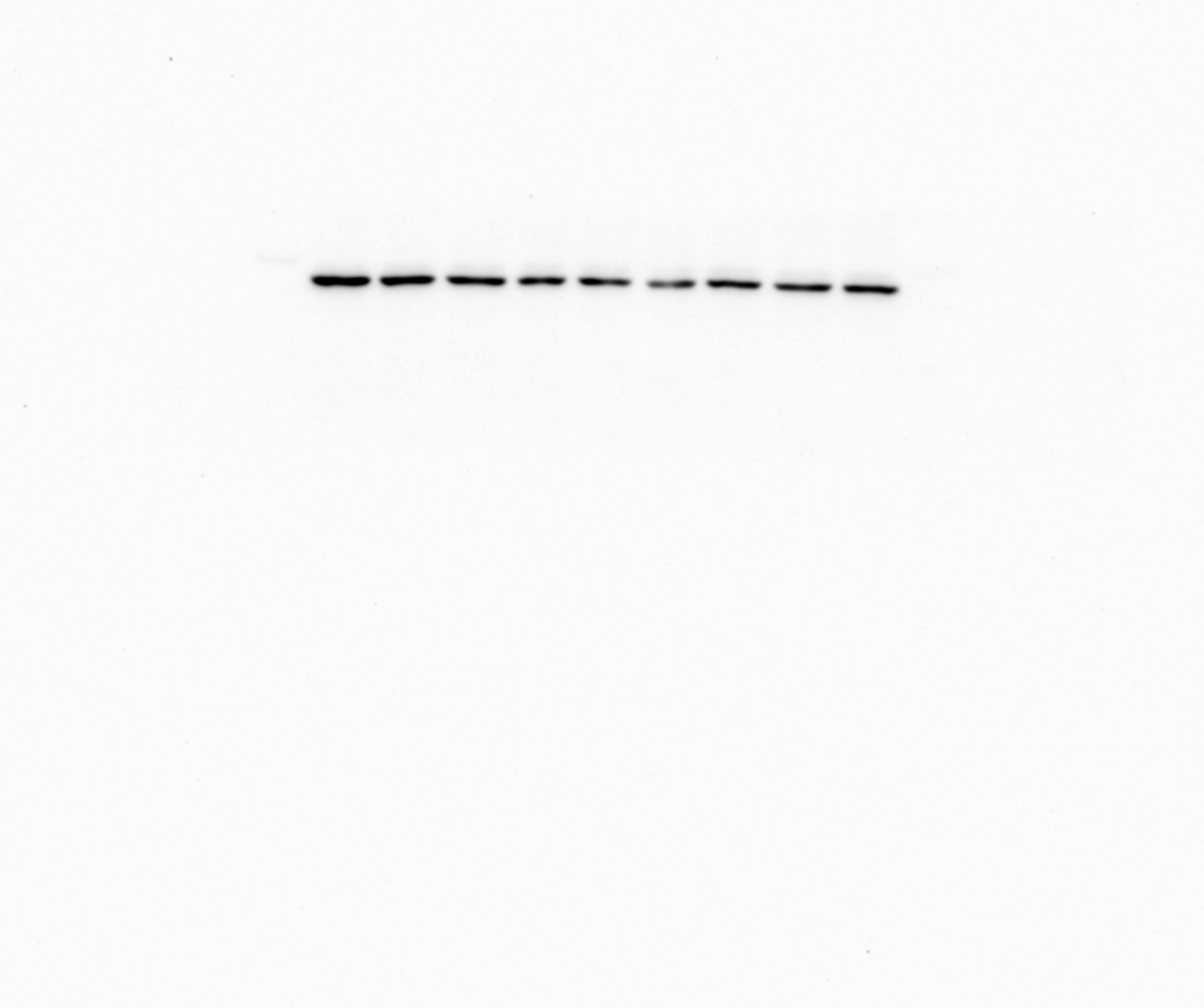

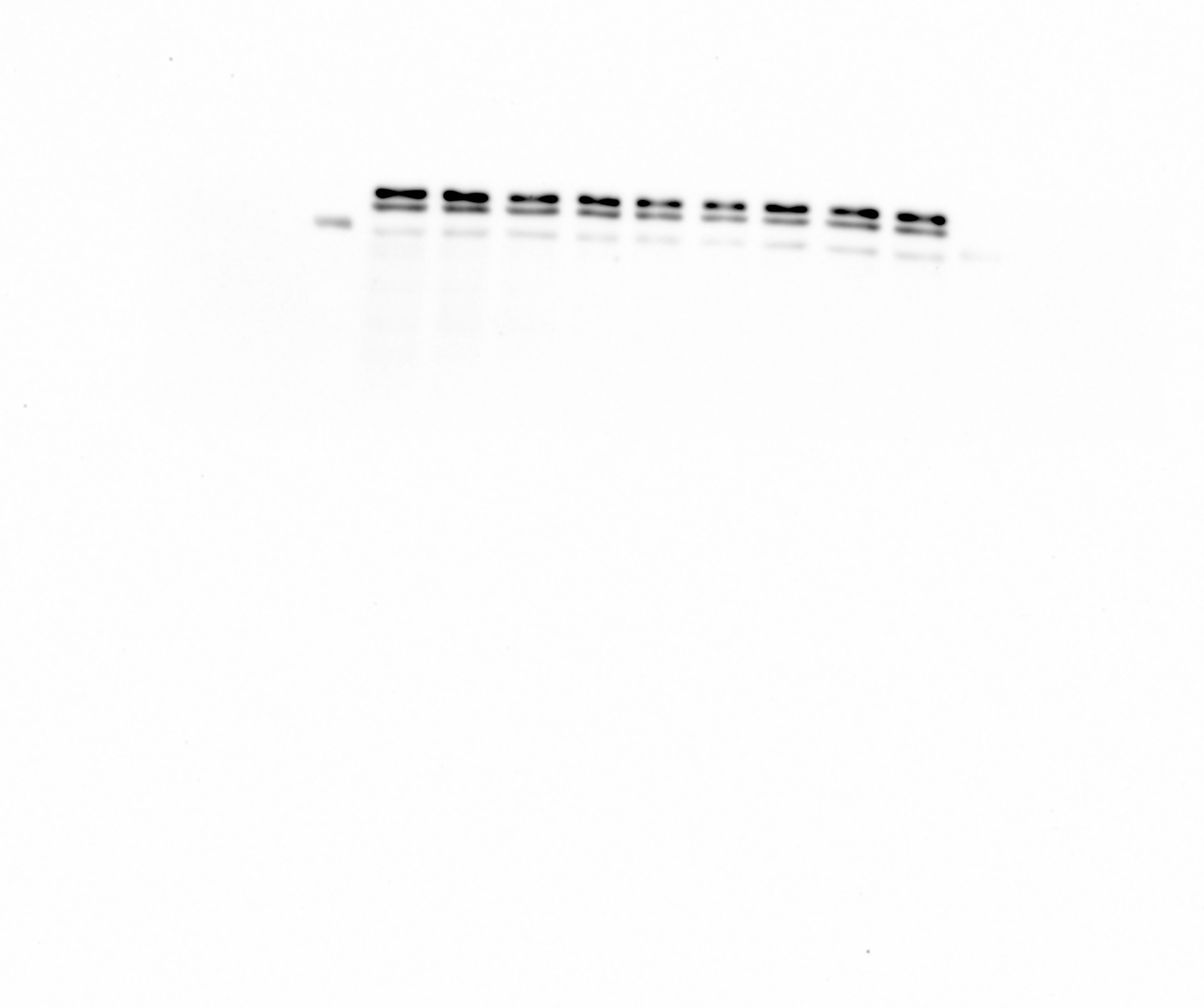


**Ctrl+CD MPTP+CD MPTP+KD**

**A**

**~85 KD-**

**~100 KD-**

**Nrf2**

**P-PI3K**


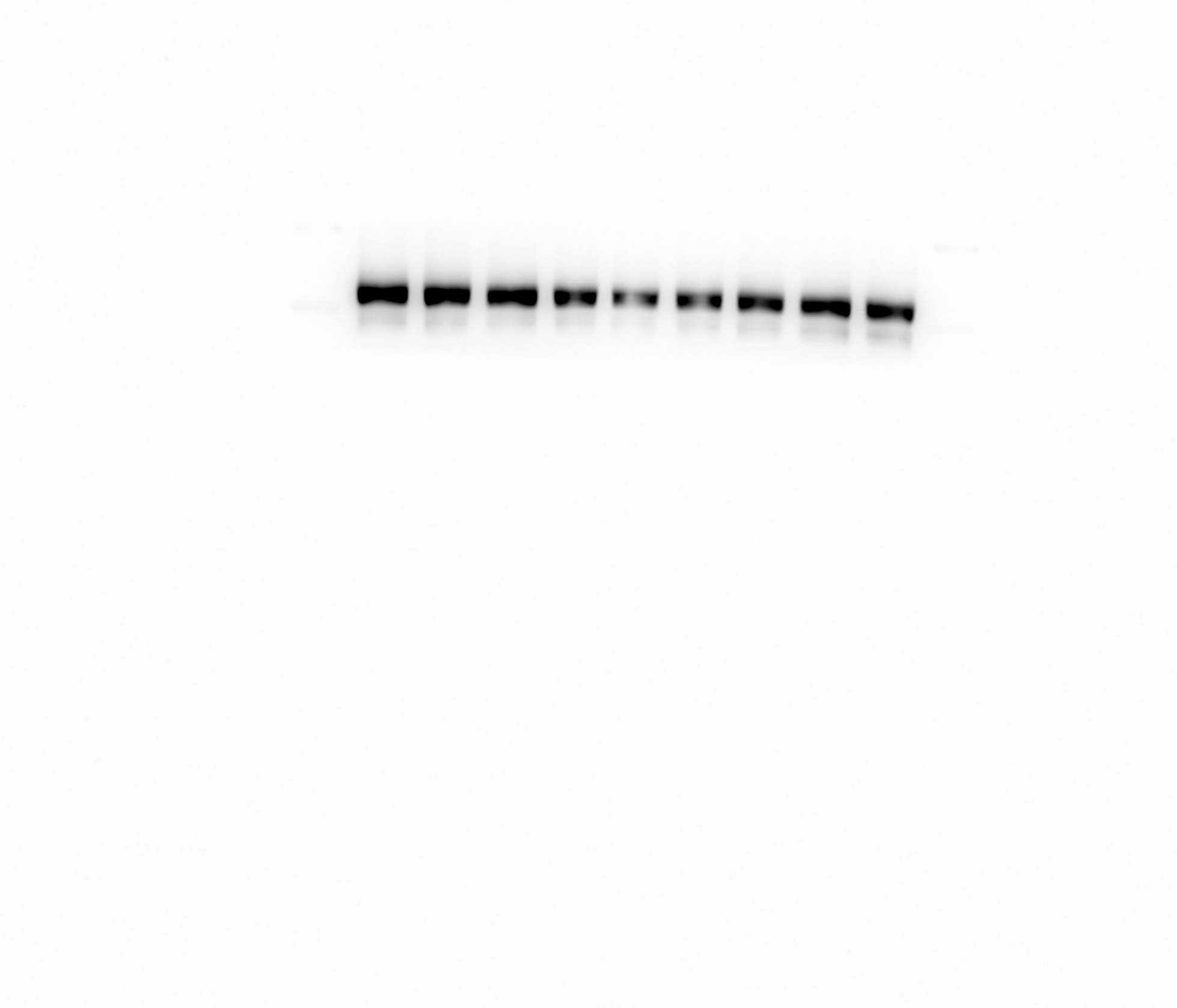

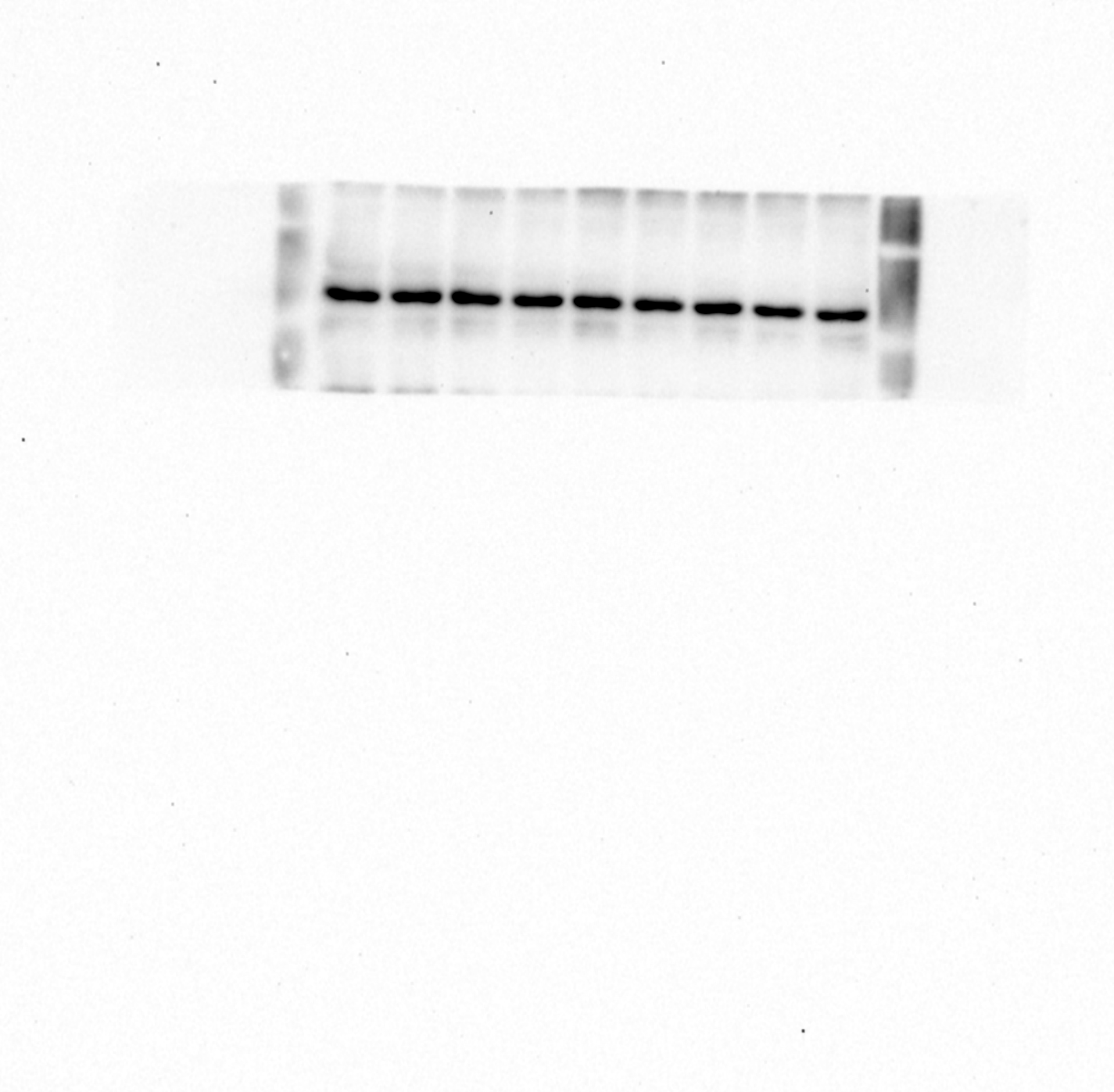


**~110 KD-**

**PI3K**

**~60 KD-**

**P-Akt**


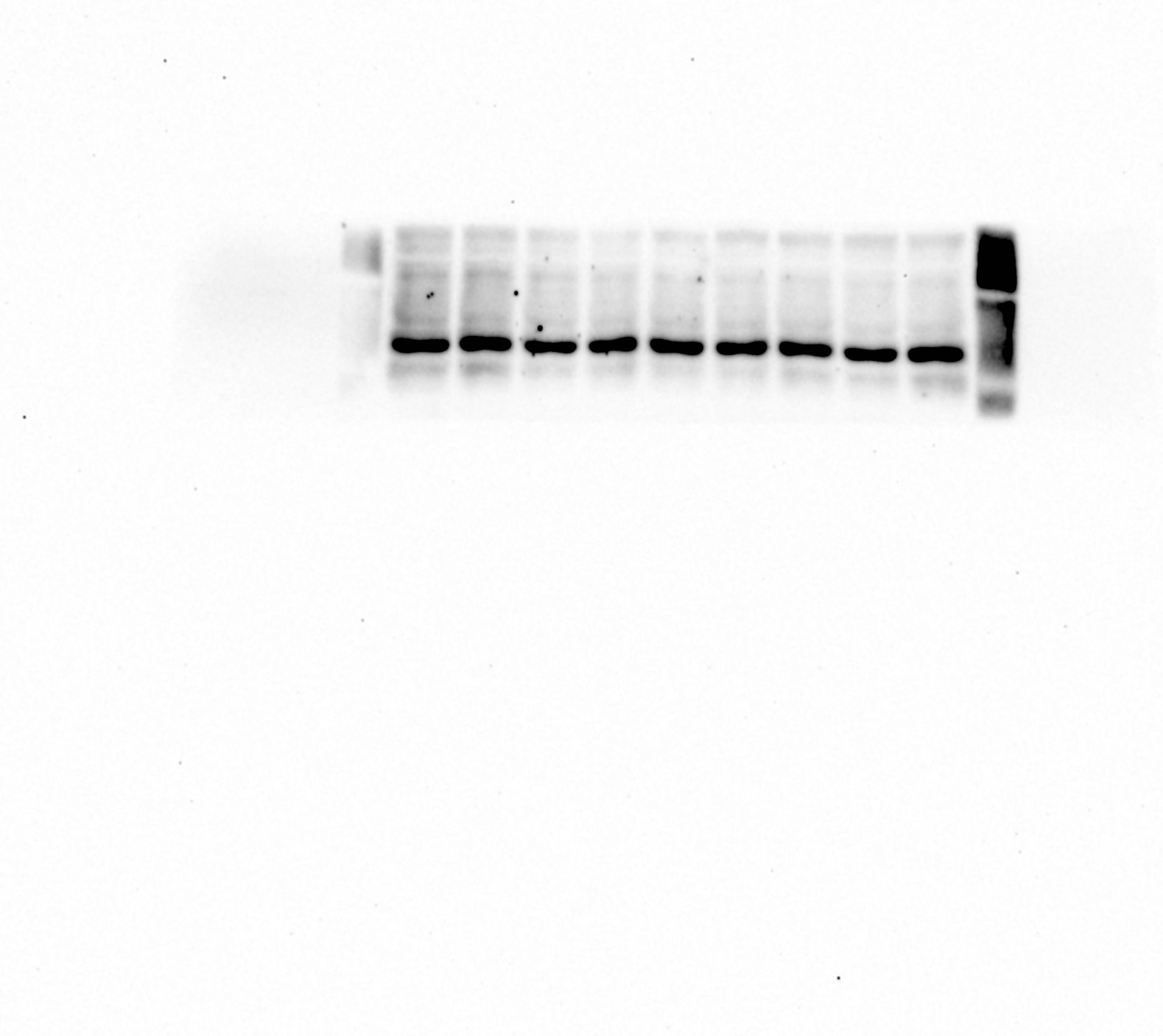


**~70 KD-**

**~60 KD-**

**Akt**


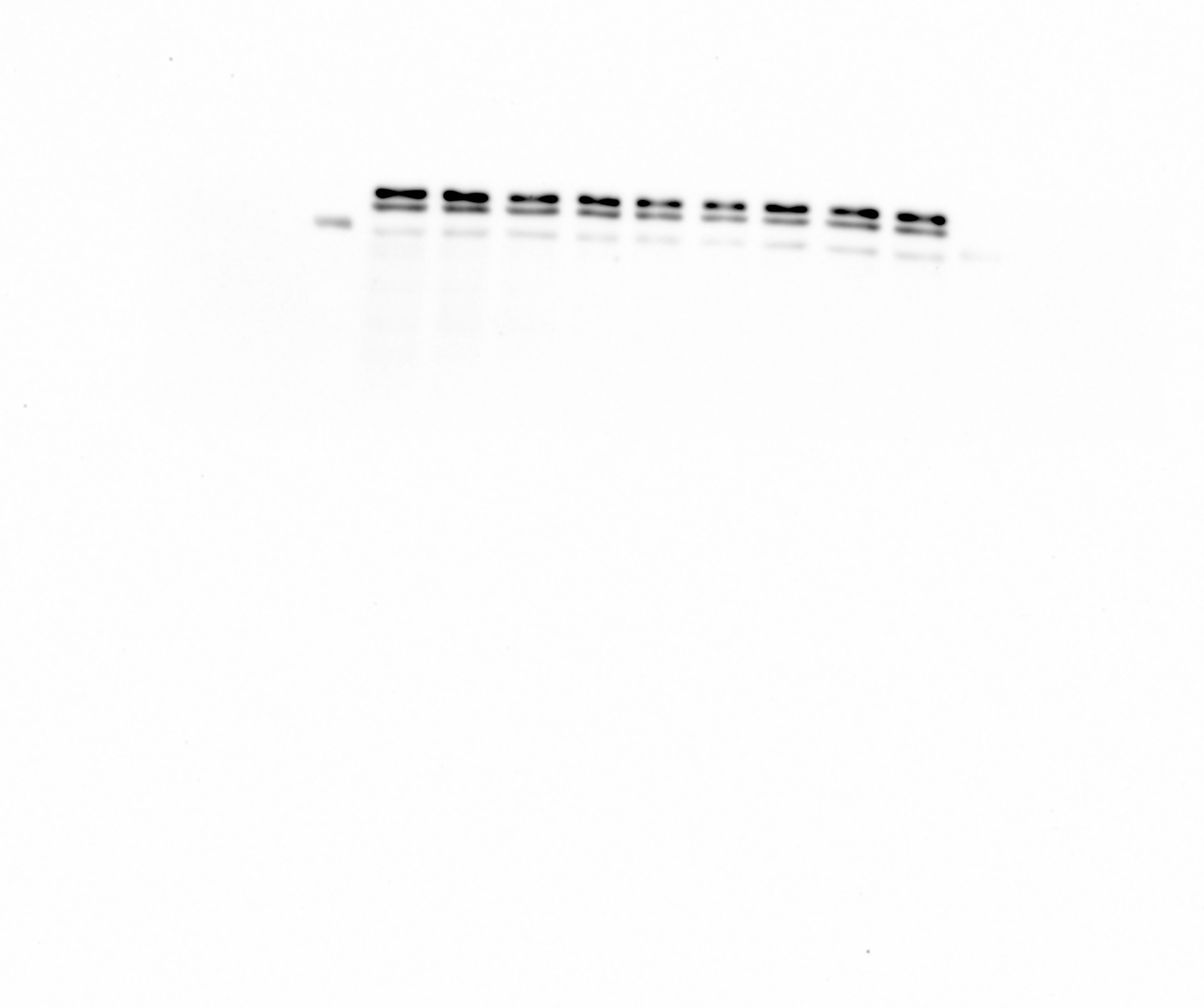


**~100 KD-**

**Nrf2**

**Nrf2**

**GAPDH**


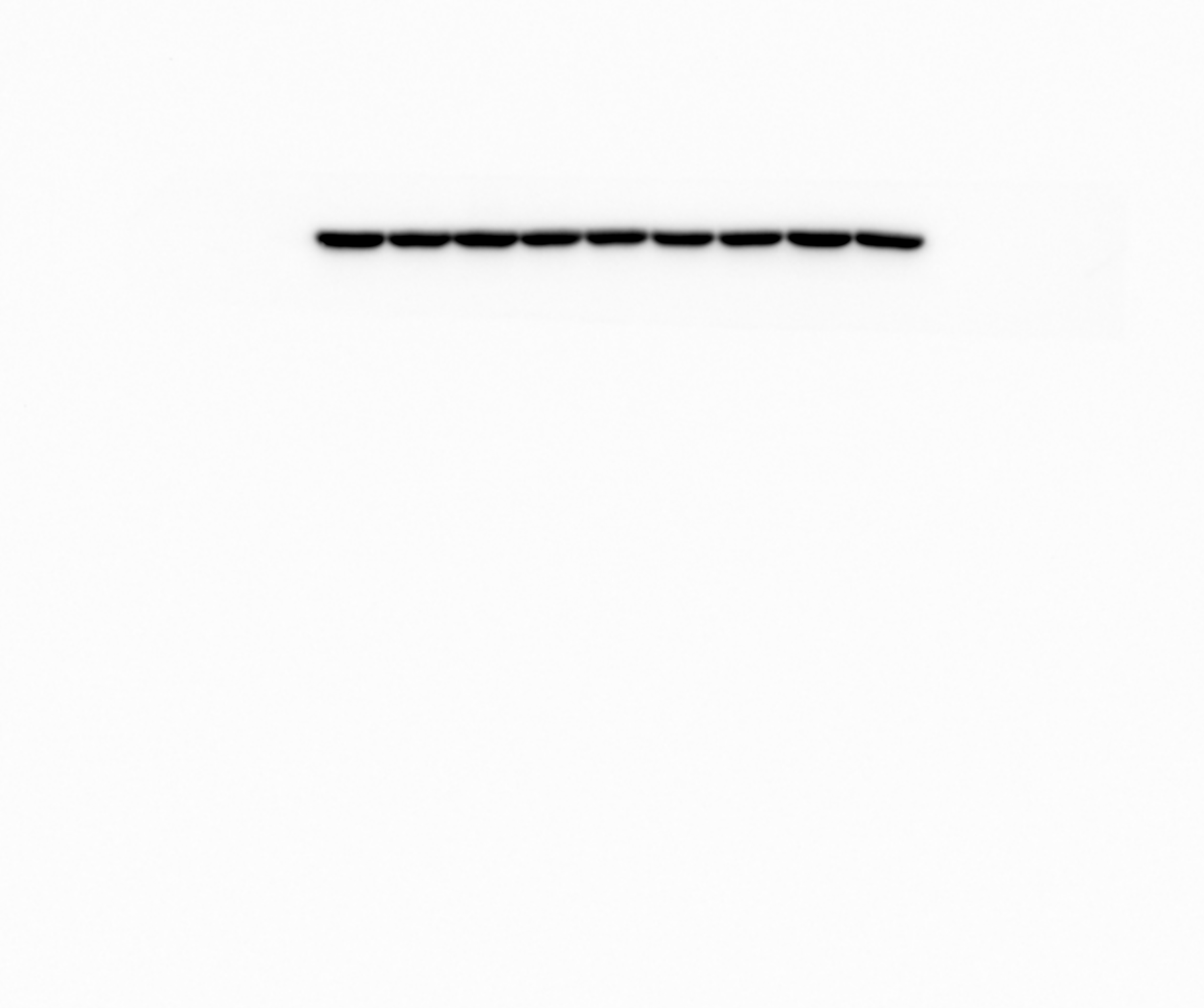


**~37 KD-**

**GAPDH**


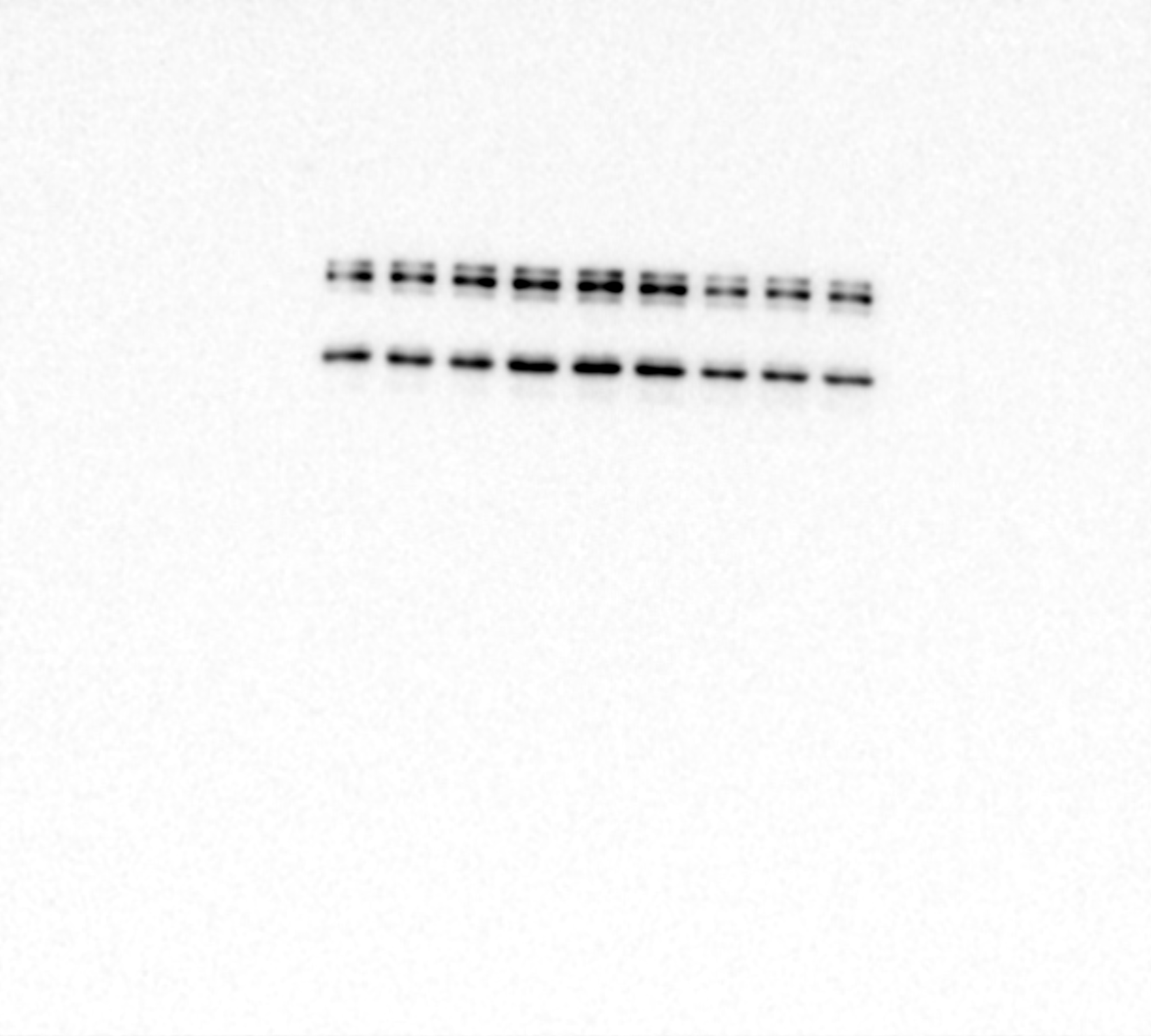

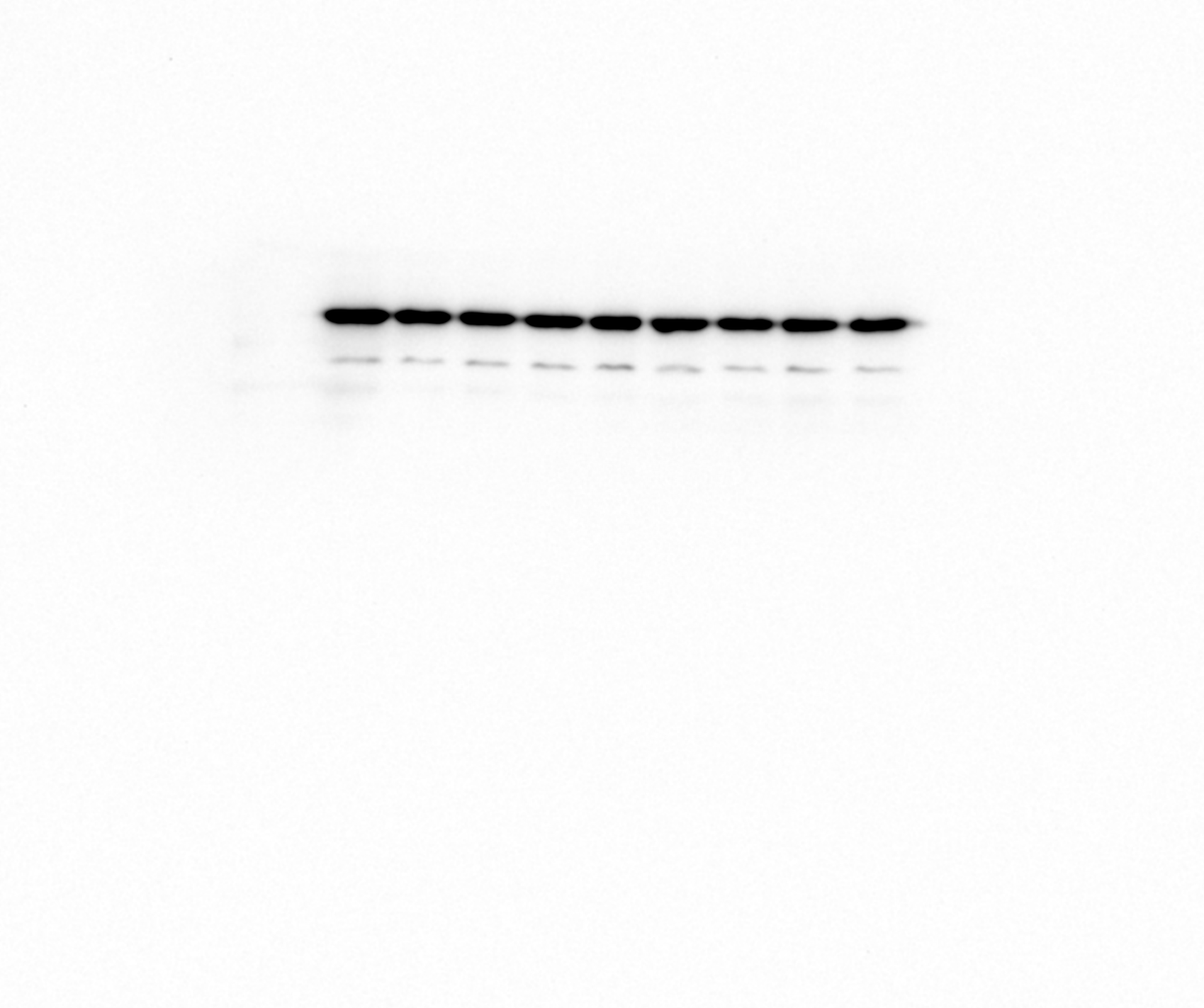

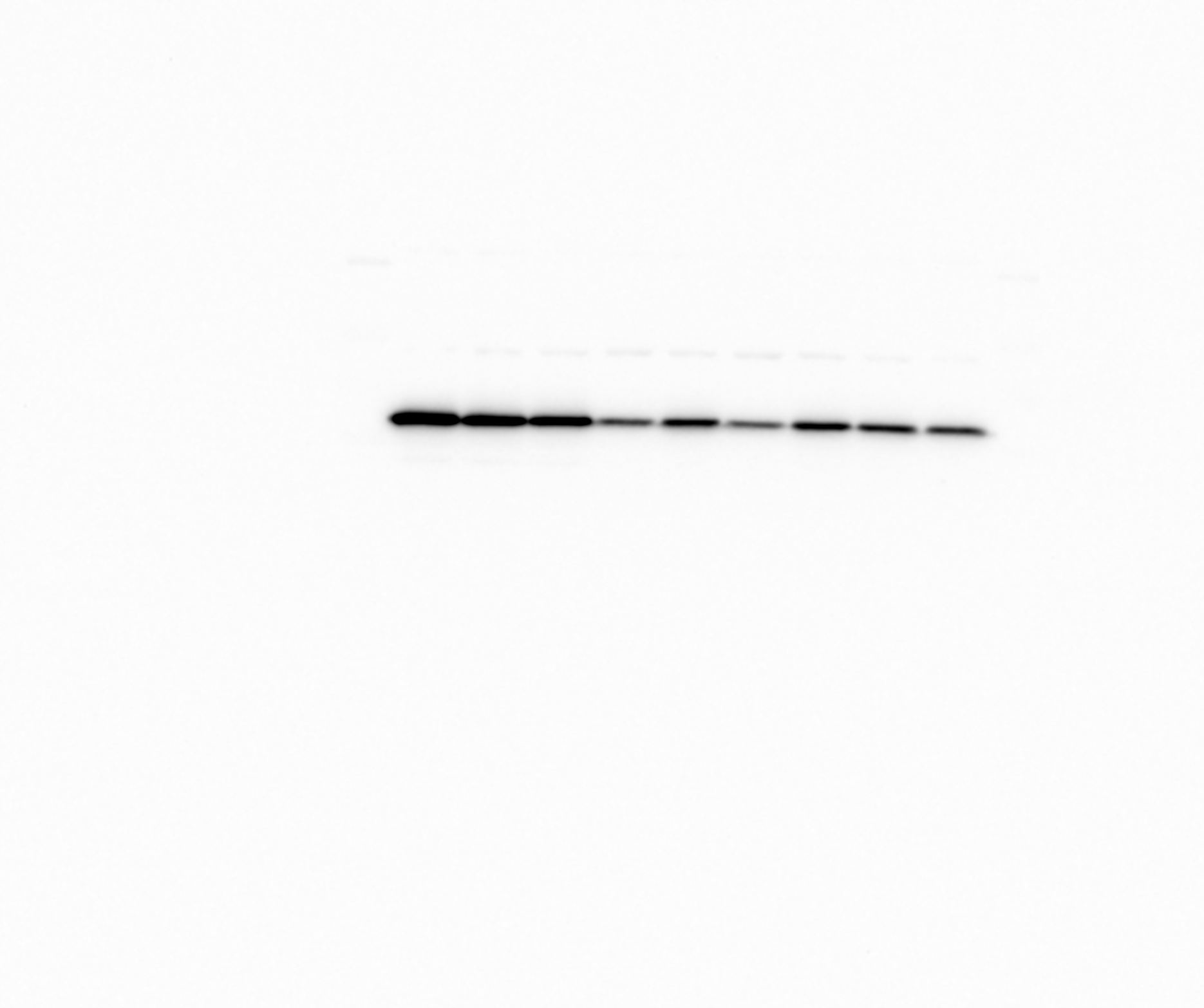


**Ctrl+CD MPTP+CD MPTP+KD**

**C**

**~26 KD-**

**GAPDH**

**~37 KD-**

**Bax**

**~20 KD-**

**Bcl-2**

**35KD-**
