# Supplementary material for: Neuroprotective effect of a medium-chain triglyceride ketogenic diet on MPTP-induced Parkinson’s disease mice: a combination of transcriptomics and metabolomics in the substantia nigra and fecal microbiome
Source: Cell Death Discov. 2023 Jul 17;9:251. doi: 10.1038/s41420-023-01549-0 (PMC10352270; doi:10.1038/s41420-023-01549-0)
Supplement: Supplementary file 2 — Supplementary figure legends [file 41420_2023_1549_MOESM2_ESM.docx]

**sFigure 1. MCT-KD exerted no observable toxicity in major organs.** Representative graphs for hematoxylin and eosin staining in major organs (including liver, heart, kidney, lung, spleen and colon) from Control + CD group, MPTP + CD group, MPTP +KD group, Control + KD group. There were no detectable pathological changes in the major organs derived from MPTP mice treated with MCT-KD. Black arrows indicate pathological injury. Scale bars, 100 µm.

**sFigure 2. Relative abundance of fecal pellets metabolites in MPTP-treated mice.** Relative metabolities abundance of differential fecal pellets metabolites between Ctrl + CD, MPTP + CD, MPTP + MCT-KD groups.

**sFigure 3. Correlation analysis of intestinal microbe and metabolites**. The co-variance between the bacterial species abundance and the abundance of metabolites. Red color of *p* -value indicates positive correlation, whereas blue color negative correlation.

**sFigure 4. Correlation analysis of differential metabolites and differential genes in midbrain substantia nigra**. The co-variance between the abundance of differentially expressed genes and the abundance of metabolites. Red color of *p* -value indicates positive correlation, whereas blue color negative correlation.
